# Circadian rhythm-related factors of PER and CRY family genes function as novel therapeutic targets and prognostic biomarkers in lung adenocarcinoma

**DOI:** 10.18632/aging.204386

**Published:** 2022-11-16

**Authors:** Chin-Chou Wang, Wei-Hsun Lin, Su-Chi Ku, Wan-Jou Shen, Hoang Dang Khoa Ta, Gangga Anuraga, Fang-Wen Liu, Chiu-Fan Shen, Shu-He Wang, Chia-Chen Yang, Chih-Yang Wang, Wei-Jan Wang

**Affiliations:** 1 Department of Biological Science and Technology, China Medical University, Taichung 40676, Taiwan; 2 Divisions of Pulmonary and Critical Care Medicine, Department of Internal Medicine, Kaohsiung Chang Gung Memorial Hospital, Chang Gung University College of Medicine, Kaohsiung 83301, Taiwan; 3 Department of Respiratory Therapy, Kaohsiung Chang Gung Memorial Hospital, Chang Gung University College of Medicine, Kaohsiung 83301, Taiwan; 4 Department of Respiratory Care, Chang Gung University of Science and Technology, Chiayi 613016, Taiwan; 5 Graduate Institute of Cancer Biology and Drug Discovery, College of Medical Science and Technology, Taipei Medical University, Taipei 11031, Taiwan; 6 Ph.D. Program for Cancer Molecular Biology and Drug Discovery, College of Medical Science and Technology, Taipei Medical University and Academia Sinica, Taipei 11031, Taiwan; 7 School of Medicine, College of Medicine, Taipei Medical University, Taipei 11031, Taiwan; 8 Graduate Institute of Biomedical Sciences, College of Medicine, China Medical University, Taichung 40402, Taiwan; 9 Department of Statistics, Faculty of Science and Technology, Universitas PGRI Adi Buana, Surabaya, East Java 60234, Indonesia; 10 TMU Research Center of Cancer Translational Medicine, Taipei Medical University, Taipei 11031, Taiwan; 11 Research Center for Cancer Biology, China Medical University, Taichung 40676, Taiwan

**Keywords:** circadian rhythm, lung adenocarcinoma, biomarker, PER, CRY

## Abstract

The period (*PER*) and cryptochrome (*CRY*) families play critical roles in circadian rhythms. The imbalance of circadian factors may lead to the occurrence of cancer. Expressions of *PER* and *CRY* family members decrease in various cancers. Nevertheless, expression levels, genetic variations, and molecular mechanisms of *PER* and *CRY* family members in lung adenocarcinoma (LUAD) and their correlations with prognoses and immune infiltration in LUAD patients are still unclear. In this study, to identify their biological functions in LUAD development, comprehensive high-throughput techniques were applied to analyze the relationships of expressions of *PER* and *CRY* family members with genetic variations, molecular mechanisms, and immune infiltration. The present results showed that transcription levels of *PER1* and *CRY2* in LUAD were significantly downregulated. High expression levels of *PER2*, *PER3*, *CRY1*, and *CRY2* indicated longer overall survival. Some cancer signaling pathways were related to *PER* and *CRY* family members, such as cell-cycle, histidine metabolism, and progesterone-mediated oocyte maturation pathways. Expressions of *PER* and *CRY* family members significantly affected the infiltration of different immune cells. In conclusion, our findings may help better understand the molecular basis of LUAD, and provide new perspectives of *PER* and *CRY* family members as novel biomarkers for LUAD.

## INTRODUCTION

According to a report provided by cancer statistics 2022, the most commonly diagnosed cancer is lung cancer, which is also the leading cause of cancer deaths in the world [1]. Lung cancer is divided into two categories, namely small-cell lung cancer (SCLC) and non-SCLC (NSCLC). NSCLC accounts for more than 85% of all lung cancers, and it is subdivided into lung adenocarcinoma (LUAD), squamous cell lung cancer, and large-cell lung cancer. LUAD accounts for the highest proportion of NSCLC cases, and finding efficacious treatments for LUAD is one of the main research goals of researchers. In recent years, although the pathogenesis of LUAD and new treatment strategies have been discovered, LUAD is still one of the most aggressive and fatal types of lung cancer, with low 5-year overall survival (OS) rates. Therefore, finding novel biomarkers for LUAD is desperately in demand [2–6]. Recently, it was proven that circadian rhythms act as a crucial factor causing cancer, as an abnormal lifestyle may disrupt and break natural circadian rhythms [7–9]. Circadian rhythms can regulate cell proliferation, cell death, DNA repair, and metabolic functions [10, 11]. Changes in circadian rhythms may lead to loss of these regulatory functions and further lead to the development of cancer. The suprachiasmatic nucleus (SCN) plays an important role in the circadian rhythms of mammals [12], and it uses a molecular oscillator to maintain clock oscillation at a normal pace [13]. The molecular oscillator consists of interacting molecular loops, composed of positive elements including circadian locomotor output cycles kaput (CLOCK) and brain and muscle ARNT-like 1 (BMAL1), and negative elements including period (PER) circadian regulators and cryptochrome (CRY) circadian regulators [14]. In addition, the core oscillatory mechanism of the SCN begins from the heterodimer CLOCK/BMAL1 complex binding to E-box elements in their regulatory regions and activating target genes to initiate transcription of *PER* and *CRY* [15], which are translated into proteins and accumulate in the cytoplasm during the daytime. After the proteins have accumulated to a certain level, the PER and CRY proteins form a complex that is then translocated into nuclei to inhibit their own transcription at night [16]. Afterward, the PER and CRY proteins are gradually phosphorylated at night, and degraded by the proteasome after being ubiquitinated by a specific E3 ligase [17–19]. The SCN can adjust the circadian rhythm stage by receiving photic and non-photic signals [20].

PER and CRY are important negative regulators of circadian rhythms [21]. Studies showed that CRY-deficient mice produce angiopoietin-like protein 2 expression [22], and CRY was proven to be related to insulin-like growth factor (IGF), which plays important roles in cell proliferation, growth, and cancer [23]. Similarly, according to clinicopathological features, *PER1*, *PER2*, and *PER3* are obviously methylated in breast cancer patients [24]. *PER* expression in colorectal cancer cells is also significantly lower than that of normal colorectal mucosal cells [25]. Recent studies discovered the mechanisms by which circadian factors affect certain cancers. For example, melatonin can inhibit the activity and the growth of prostate cancer cells by upregulating PER2 [26]. CLOCK/BMAL1/PER/CRY were also found to alter the c-Myc/p21 and Wnt/β-catenin pathways to varying degrees to affect DNA damage [27]. In addition, circadian rhythms are thought to be related to the immune system, as CRY can affect some key inflammatory pathways such as nuclear factor (NF)-κB [28]. Despite the fact that there is research on PER and CRY in various cancers, current studies have not fully elucidated expression levels, gene variations, molecular functions, or their relationships with prognoses and immune infiltrations in LUAD.

Few previous studies reported the roles of PER and CRY in lung cancer. In particular, interactions and pathways between all *PER* and *CRY* family members and related molecules in tumorigenesis are still unclear. Multiple microarray and sequencing technologies have enhanced the ability of robust computational algorithms to rapidly analyze biomedical data [29–34]. Examining gene expressions and employing appropriate algorithms are thought to be able to help us understand the respective functions of *PER* and *CRY* in lung cancer development. In this study, different bioinformatics databases were incorporated to analyze various *PER* and *CRY* family members in LUAD to understand expressions of these factors, molecular functions such as proliferation or tumorigenesis, and their impacts on OS, genetic changes, immune infiltration, and immune checkpoints in LUAD, which would help identify whether *PER* and *CRY* are suitable biomarkers for precision treatment and detection of LUAD.

## RESULTS

### Transcriptional levels of PER and CRY family members in LUAD patients

**Figure 1:**
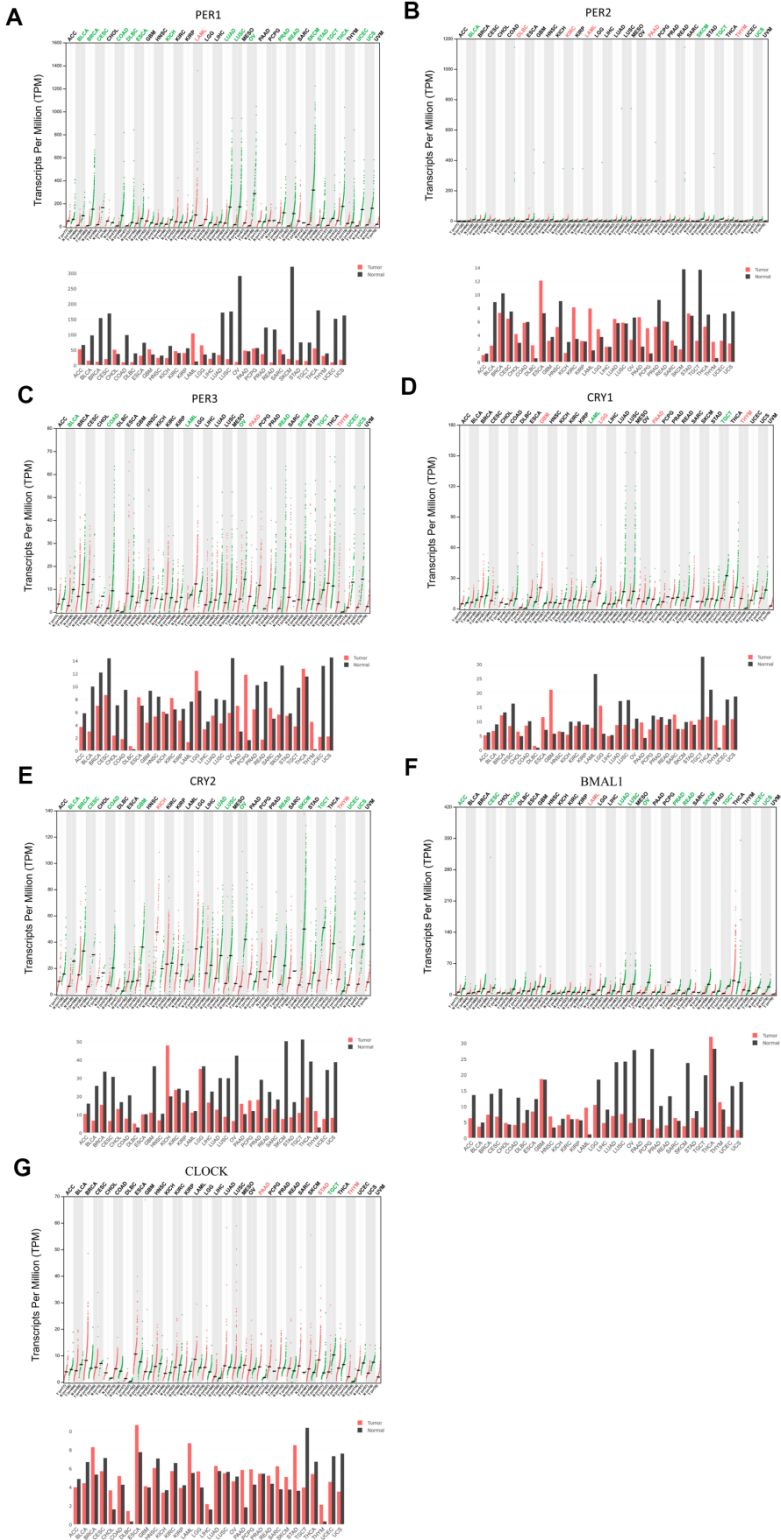
**Expression levels of period (*PER*) family (*PER1*, *PER2*, and *PER3*), cryptochrome (*CRY*) family (*CRY1* and *CRY2*), and other circadian factors such as *BMAL1* and *CLOCK* in different types of cancer (GEPIA).** (**A**–**G**) This figure shows mRNA expression levels of the *PER* and *CRY* families of circadian factors in different cancer tissues. If a cancer had significant overexpression of a gene, the name of the cancer is shown in red. Conversely, if a cancer had a significantly low expression of the gene, green color indicates the name of the cancer.

Circadian factors are widely expressed by mammalian cells, but have different expression levels in different tumor tissues. The UALCAN database showed expressions of circadian factors, including *PER* and *CRY* family members, *BMAL1* (*ARNTL*), and *CLOCK*, with differential expression levels between different types of cancers and normal tissues (Supplementary Figure 1). GEPIA was used to understand the messenger (m)RNA expressions of circadian rhythm-related factors in the *PER* family (*PER1*, *PER2*, and *PER3*), *CRY* family (*CRY1* and *CRY2*), *BMAL1*, and *CLOCK* in different types of cancer (Figure 1).

**Figure 2:**
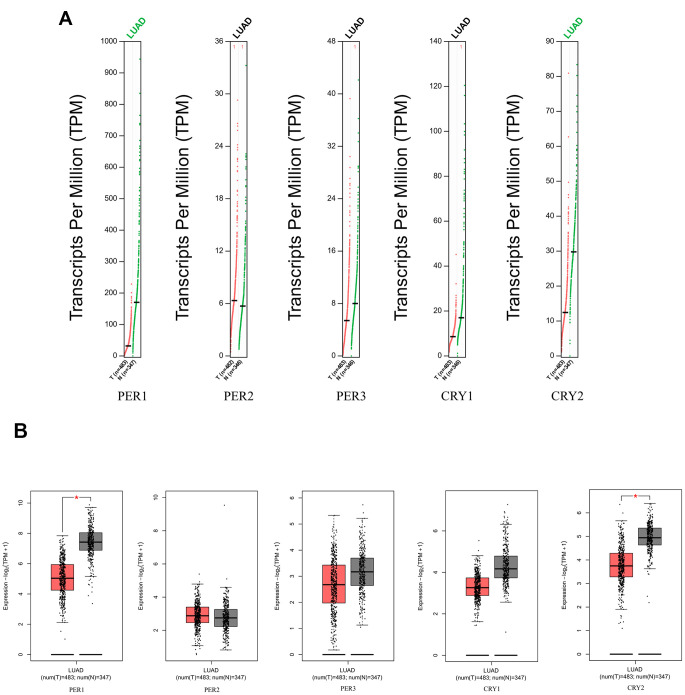
**Expression levels of *PER* (period) and *CRY* (cryptochrome) family members in lung adenocarcinoma (LUAD) patients (GEPIA2), with the q-value cutoff set to 0.01.** (**A**) mRNA expressions of *PER1*, *PER2*, *PER3*, *CRY1*, and *CRY2* in LUAD (red) and normal lung tissues (green). The name of a cancer in green indicates that the mRNA expression of the gene in normal tissues was significantly higher than in cancer tissues. (**B**) mRNA expressions of *PER1*, *PER2*, *PER3*, *CRY1*, and *CRY2* in LUAD (red) and normal lung tissues (gray). Red stars indicate significant differences in the data.

The Oncomine database displays mRNA expression levels of *PER* and *CRY* family members in different types of tumors and normal tissue samples (Supplementary Figure 2). The Oncomine analysis showed that transcription levels of both *PER* and *CRY* family members were downregulated in lung cancer patients. Transcription levels of all members of the *PER* and *CRY* families were significantly lower than those in normal tissues (Supplementary Table 1). In the LUAD dataset of Bhattacharjee Lung [35], Stearman Lung [36], Landi Lung [37] and Okayama Lung [38], transcriptional levels of *PER1* in tumors were lower than those in normal samples with -5.555- (*p* = 3.35E-5), -1.717- (*p* = 1.38E-7), -2.125- (*p* = 8.29E-19), and -2.148-fold changes (*p* = 1.77E-8), respectively. In the Su Lung dataset [39], expressions of *PER1*, *PER2*, *CRY1*, and *CRY2* were -1.861- (*p* = 6.23E-7), -1.803- (*p* = 1.29E-7), -2.058- (*p* = 1.39E-6), and -3.450-fold lower (*p* = 2.47E-5) in LUAD than in normal samples. In the Hou Lung [40] dataset, *PER3*, *CRY1*, and *CRY2* were significantly lower than in normal tissues in LUAD patients with respective fold changes of -2.024 (p = 1.05E-9), -1.702 (p = 5.10E-13), and -1.836 (p = 9.60E-13). In GEPIA2, *PER1* and *CRY2* were also found to have higher expressions in normal lung tissues than in LUAD tissues (Figure 2).

**Figure 3:**
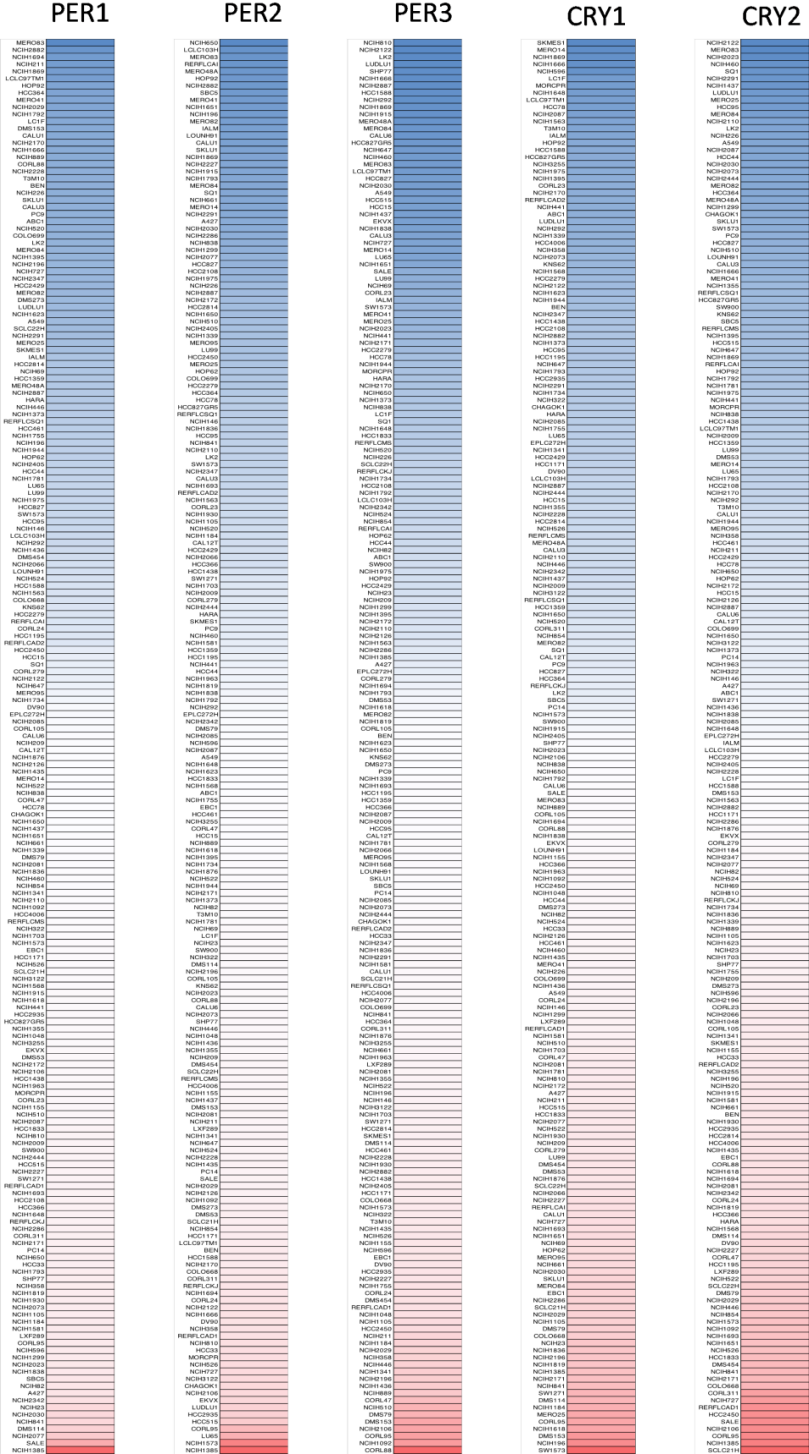
**Gene expression levels of *PER* (period) and *CRY* (cryptochrome) family members when screening 198 lung cancer cell lines (CCLE database).*** PER1*, *PER2*, *PER3*, *CRY1*, and *CRY2* expression levels were differentiated into five columns. The blue blocks indicated under-expression, whereas the red blocks represented overexpression.

Furthermore, we used the CCLE to analyze mRNA expression levels of *PER1*, *PER2*, *PER3*, *CRY1*, and *CRY2* in lung cancer cell lines in current lung cancer research. Then, the CCLE analysis was presented to reveal transcriptomic levels of *PER* and *CRY* family members in 198 lung cancer cell lines (Figure 3). Comprehensive results are clearly described in Figure 3 and show mRNA expression levels of *PER1*, *PER2*, *PER3*, *CRY1*, and *CRY2* in different lung cancer cell lines. Blue represents a lowly expressed gene in a cell line, while red indicates a highly expressed gene in a cell line. The shade of the color represents the degree of high or low expression.

### Prognostic values of PER and CRY family members in LUAD patients

**Figure 4:**
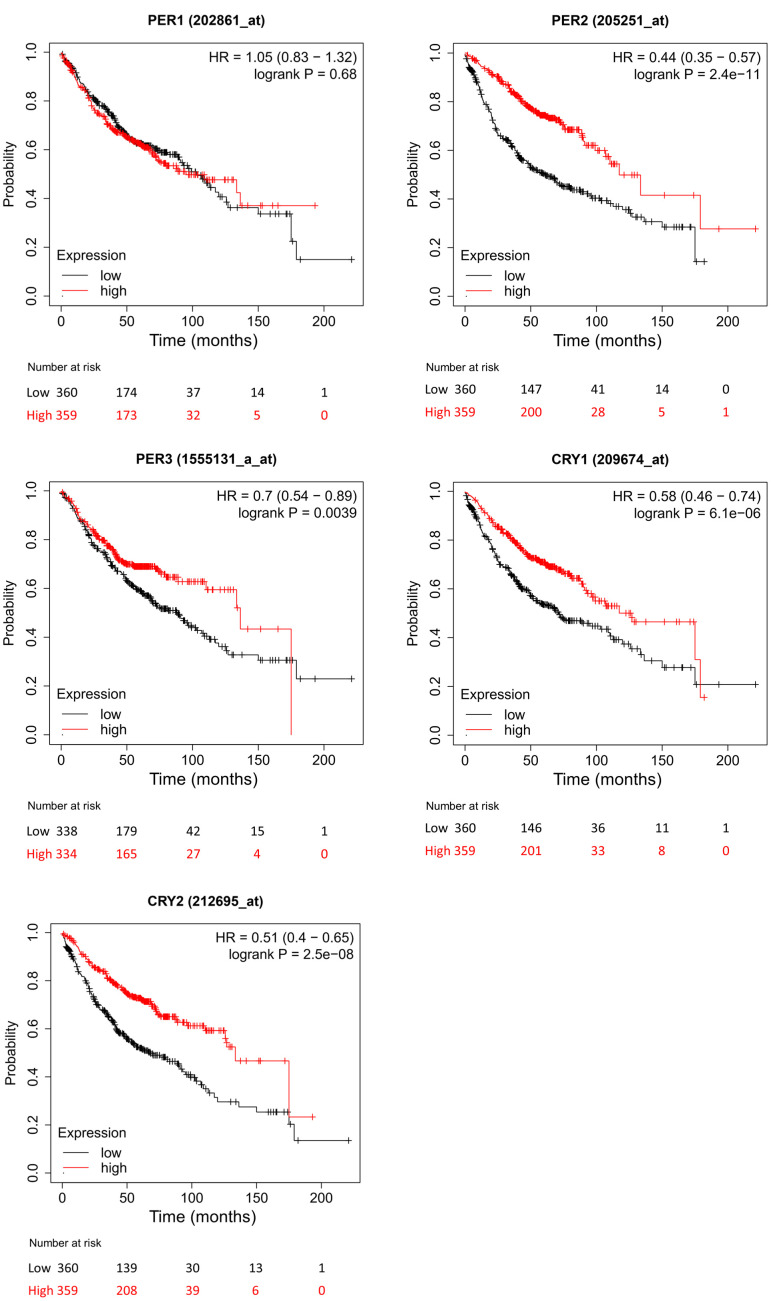
**Different expressions of *PER* (period) and *CRY* (cryptochrome) family members in lung adenocarcinoma (LUAD) patients in the overall survival (OS) curve (using the Kaplan-Meier plotter).** The red line represents the survival rate curve of patients with LUAD who expressed the gene, and the black line represents the survival rate curve of LUAD patients who did not express the gene.

To evaluate different transcription levels of *PER* and *CRY* family members in the LUAD, we used the KM plotter to analyze correlations of *PER* and *CRY* family members with clinical results. OS results showed that high expression levels of *PER2*, *PER3*, *CRY1*, and *CRY2* were significantly related to longer OS in LUAD patients (Figure 4). In contrast, *PER1* expression in LUAD patients was not significantly related to OS.

### Analysis of genetic changes and coexpressions of PER and CRY family proteins in LUAD patients

**Figure 5:**
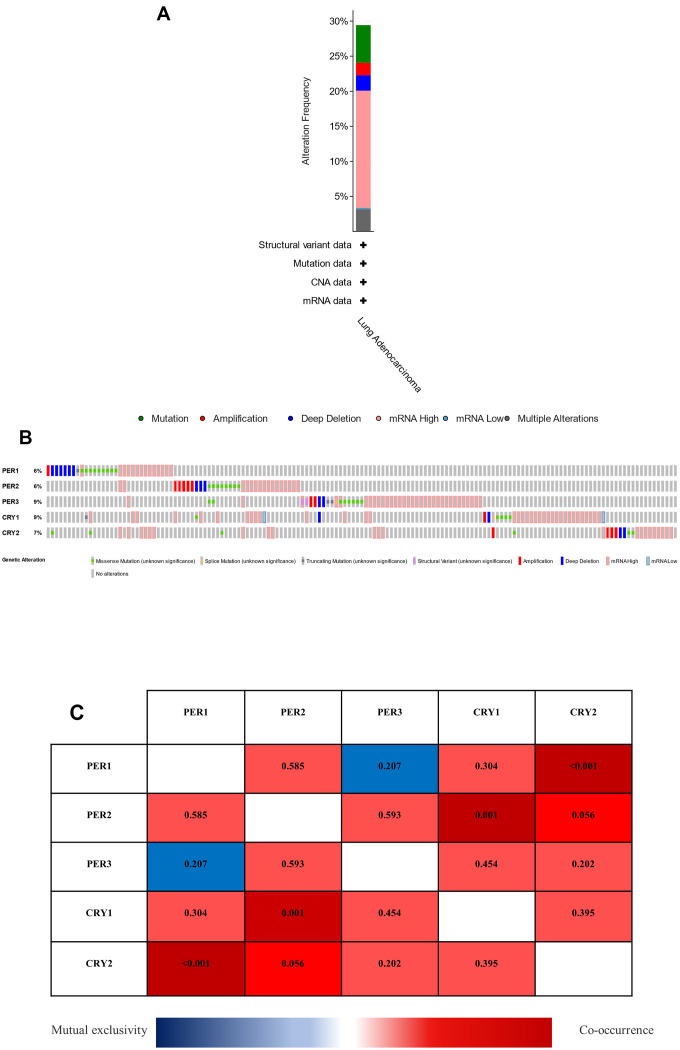
**Analysis of genetic changes, coexpressions of *PER* (period) and *CRY* (cryptochrome) in lung adenocarcinoma (LUAD) patients.** (**A**) Summary of changes in *PER* and *CRY* in LUAD patients. (**B**) Summary of OncoPrint query changes to *PER* and *CRY* family members. (**C**) Heat map of expressions of different *PER* and *CRY* family members in LUAD patients. The value in each color represents the *p* value, while red represents coexpression, and blue represents mutual exclusion.

The cBioPortal web tool was used to analyze changes in PER and CRY family proteins in LUAD patients. Among 503 cases, 148 cases (29.42%) of LUAD patients had genetic changes in *PER* and *CRY* circadian rhythm-related factors (Figure 5A and Supplementary Figure 3). TCGA dataset showed that among circadian rhythm-related factors, mutation rates were highest in *PER3* and *CRY1* (9%), followed by *CRY2* (7%), and mutation rates were lowest in *PER1* and *PER2* (6%) (Figure 5B). In addition, results showed the coexpression and mutually exclusive relationships among these genes, with only *PER1* and *CRY2*, and *PER2* and *CRY1* exhibiting statistically significant coexpression relationships (*p*<0.05). Others showed coexpression and mutual exclusion without statistical significance (*p*>0.05) possibly due to insufficient sample sizes (Figure 5C).

### Analysis of gene interactions among PER1, PER2, PER3, CRY1, and CRY2 in LUAD patients

**Figure 6:**
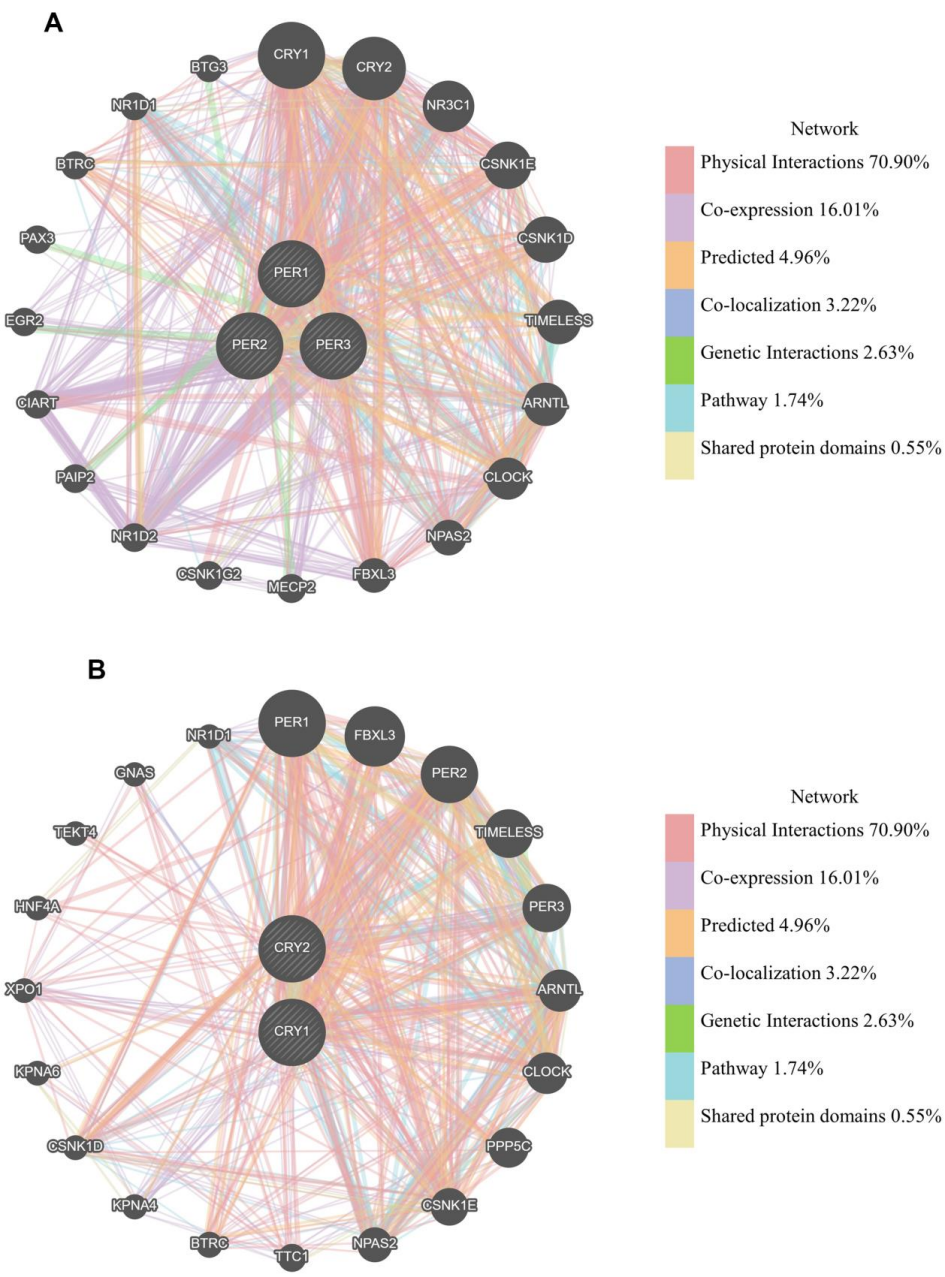
**Gene interactions among *PER1, PER2, PER3, CRY1*, and *CRY2* in lung adenocarcinoma (LUAD) patients (GeneMANIA).** (**A**) *PER* (period) family network constructed by GeneMANIA. (**B**) *CRY* (cryptochrome) family network constructed by GeneMANIA. Each node in the figure represents a gene, and the size of the node represents the intensity of the interaction. Connecting lines between nodes represent gene-gene interactions. The color of the connecting line represents the type of interaction.

From previous studies, we know that the functions of these genes are often related to regulating circadian rhythms. For instance, *CLOCK* and *ARNTL* (aryl hydrocarbon receptor nuclear translocator-like protein 1, also known as BMAL1) are positive mediators of circadian rhythms and mediate *CRY* and *PER* transcription [14]. GeneMANIA was used to construct a gene-gene interaction (GGI) network composed of *PER1*, *PER2*, *PER3*, *CRY1*, and *CRY2*, and analyze the functions that may be related to networks composed of *PER* and *CRY* family members. They were all surrounded by 20 nodes, representing genes that may have physical interactions, coexpressions, predictions, co-localizations, pathways, gene interactions, and shared protein domains with *PER* and *CRY* family members (Figure 6). In the *PER* family network (Figure 6A), the most relevant genes were *CRY1*, *CRY2*, *NR3C1* (nuclear receptor subfamily 3 group C member 1), *CSNK1E* (casein kinase 1 epsilon), *CSNK1D* (casein kinase 1 delta), *TIMELESS* (timeless circadian regulator), ARNTL, and *CLOCK*. In the *CRY* family network (Figure 6B), the most relevant genes were *PER1*, *FBXL3* (F-box and leucine-rich repeat protein 3), *PER2*, *TIMELESS*, *PER3*, *ARNTL*, *CLOCK*, and PPP5C (protein phosphatase 5 catalytic subunit).

### PPIs and functional analysis of PER and CRY family members in LUAD patients

DAVID was utilized to analyze molecular functions and Online Mendelian inheritance in man (OMIM) diseases of *PER1*-, *PER2*-, *PER3*-, *CRY1*-, and *CRY2*-related genes (Supplementary Table 2). The first five molecular functions of *PER1* (Supplementary Table 2A) were protein binding, ATP binding, microtubule-binding, single-stranded DNA binding, and microtubule motor activity. As to OMIM diseases, *PER1* was related to breast cancer and colorectal cancer (Supplementary Table 2B). The first five molecular functions of *PER2* (Supplementary Table 2C) were protein binding, poly(A) RNA binding, single-stranded DNA binding, microtubule binding, and chromatin binding. As to OMIM diseases, *PER2* was related to skin/hair/eye pigmentation 1, blond/brown hair, skin/hair/eye pigmentation 1, blue/non-blue eyes (Supplementary Table 2D). The first five molecular functions of *PER3* (Supplementary Table 2E) were protein binding, poly (A) RNA binding, MHC class II receptor activity, metal ion binding, and structural constituents of ribosomes. As to OMIM diseases, *PER3* was related to congenital dysfibrinogenemia and congenital afibrinogenemia (Supplementary Table 2F). The first five molecular functions of the *CRY* family (Supplementary Table 2G) were protein binding, single-stranded DNA-dependent ATPase activity, protein kinase binding, DNA clamp loader activity, and ATP binding [41–45]. Results demonstrated the proportions of related genes with protein-binding function were the highest with statistical significance among these five genes. Moreover, we used STRING database to separately analyze PPIs of PER and CRY family members. Supplementary Figure 4 shows the protein networks closely related to the PER (Supplementary Figure 4A) and CRY families (Supplementary Figure 4B).

In addition, to verify the detail pathway relative to *PER* and *CRY,* the database of DAVID was used to analyze and select KEGG pathways with the highest correlations with *PER1*, *PER2*, *PER3*, and *CRY* family members (Supplementary Figure 5). The most closely related KEGG pathway to *PER2* (Supplementary Figure 5A) was histidine metabolism. The KEGG pathway of progesterone-mediated oocyte maturation was the most relevant to *PER3* (Supplementary Figure 5B). The most relevant KEGG pathway to *CRY* family members (Supplementary Figure 5C) was the cell cycle like *PER1*, but the associated genes were not the same.

**Figure 7:**
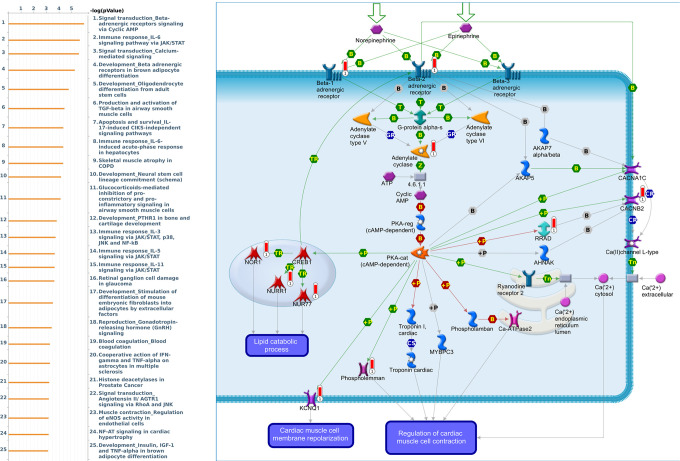
**Expression of the *PER1* signaling pathway in lung cancer (using MetaCore).** The functional analysis of “Signal transduction_Beta-adrenergic receptors signaling via cyclic AMP” was correlated with lung cancer development.

**Figure 8:**
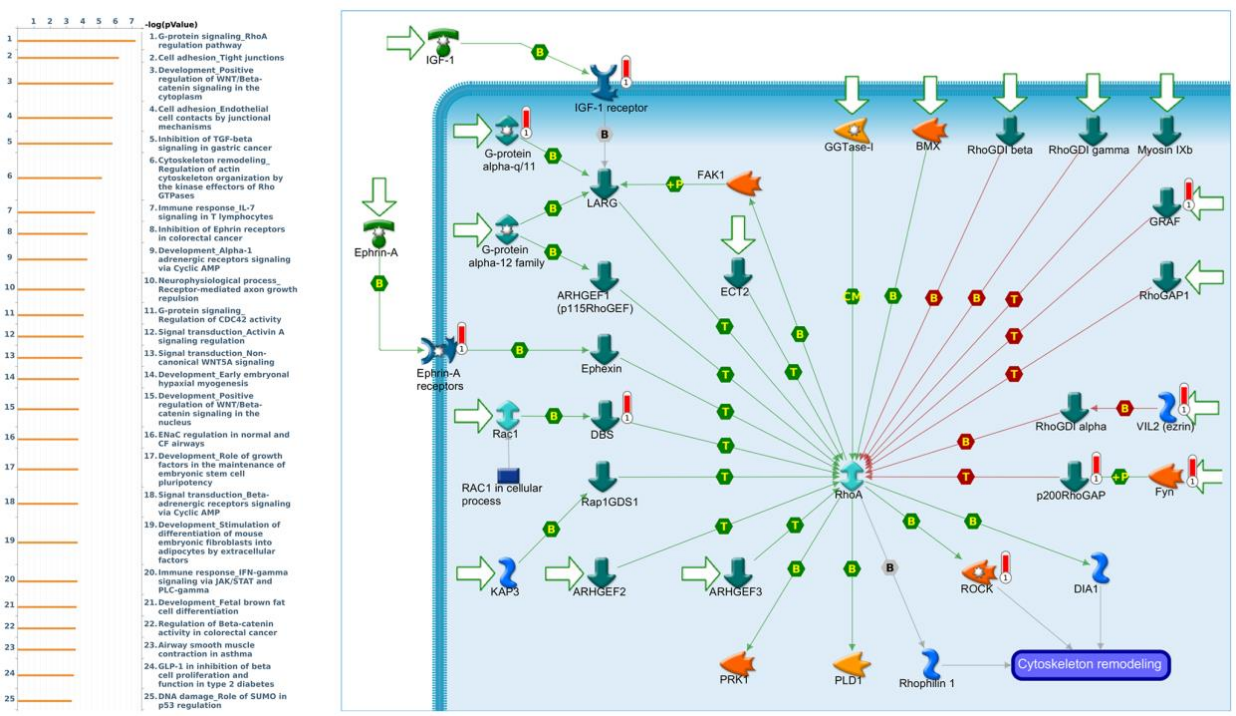
**Expression of the *PER2* signaling pathway in lung cancer (using MetaCore).** The functional analysis of the “G-protein signaling_RhoA regulation pathway” was correlated with lung cancer development.

**Figure 9:**
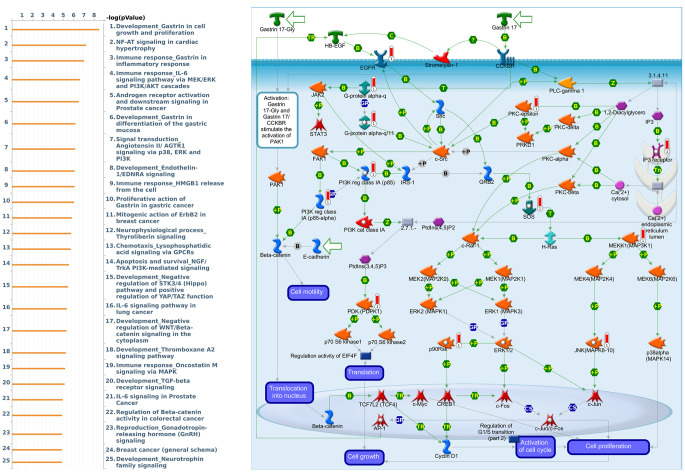
**Expression of the *PER3* signaling pathway in lung cancer (using MetaCore).** The functional analysis of “Development_Gastrin in cell growth and proliferation” was correlated with lung cancer development.

Genes coexpressed with *PER1* were correlated with “Signal transduction_Beta-adrenergic receptors signaling via cyclic AMP”, “Immune response_IL-6 signaling pathway via JAK/STAT”, and “Signal transduction_Calcium-mediated signaling” (Figure 7 and Supplementary Table 3). Genes coexpressed with *PER2* were correlated with “G-protein signaling_RhoA regulation pathway”, “Cell adhesion_Tight junctions”, and “Development_Positive regulation of WNT/Beta-catenin signaling in the cytoplasm” (Figure 8 and Supplementary Table 4). Genes coexpressed with *PER3* were correlated with “Development_Gastrin in cell growth and proliferation”, “NF-AT signaling in cardiac hypertrophy”, and “Immune response_Gastrin in inflammatory response” (Figure 9 and Supplementary Table 5).

**Figure 10:**
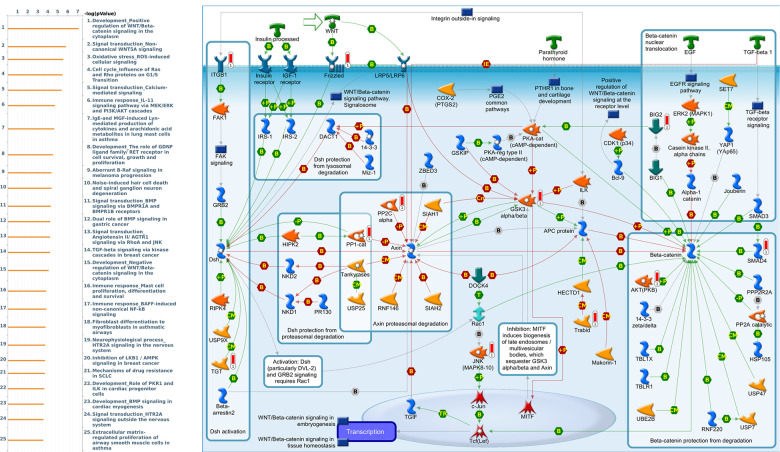
**Expression of the *CRY1* signaling pathway in lung cancer (using MetaCore).** The functional analysis of “Development_Positive regulation of WNT/Beta-catenin signaling in the cytoplasm” was correlated with lung cancer development.

**Figure 11:**
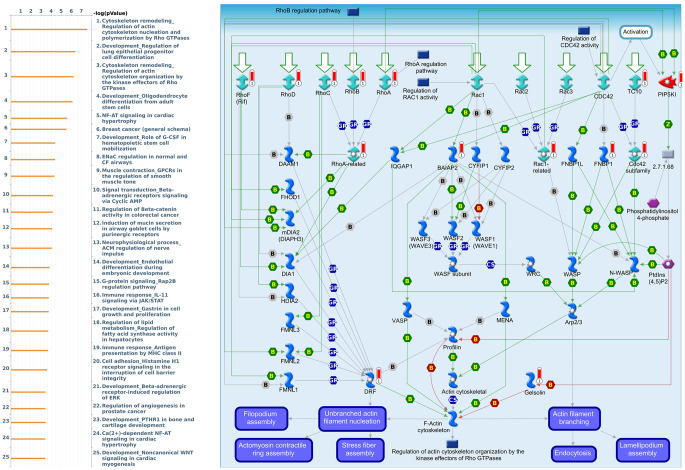
**Expression of the *CRY2* signaling pathway in lung cancer (using MetaCore).** The functional analysis of “Cytoskeleton remodeling_Regulation of actin cytoskeleton nucleation and polymerization by Rho GTPases” was correlated with lung cancer development.

Genes coexpressed with *CRY1* were correlated with “Development_Positive regulation of WNT/Beta-catenin signaling in the cytoplasm”, “Signal transduction_Non-canonical WNT5A Signaling”, and “Oxidative stress_ROS-Induced cellular signaling” (Figure 10 and Supplementary Table 6). Genes coexpressed with *CRY2* were correlated with “Cytoskeleton remodeling_Regulation of actin cytoskeleton nucleation and polymerization by Rho GTPases”, “Development_Regulation of lung epithelial progenitor cell differentiation”, and “Cytoskeleton remodeling_Regulation of actin cytoskeleton organization by the kinase effectors of Rho GTPases” (Figure 11 and Supplementary Table 7).

### Relationships between expressions of circadian factors in the PER and CRY families with immune infiltration in LUAD

**Figure 12:**
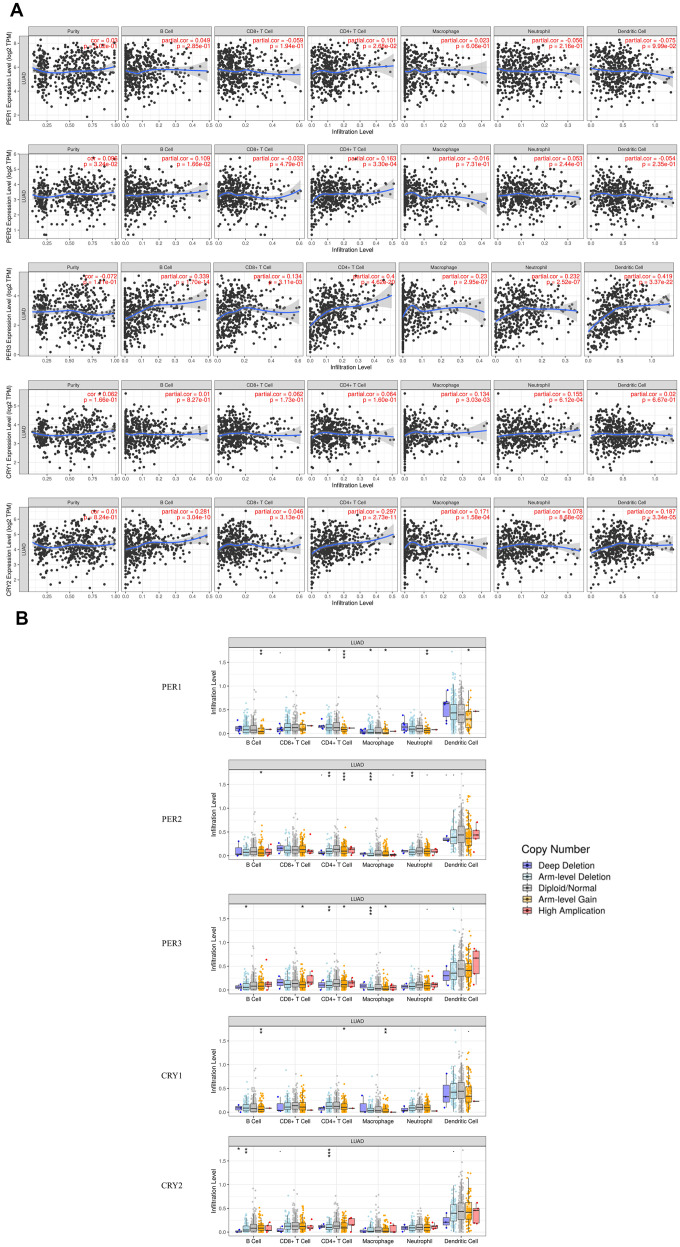
**Relationships of expressions and copy number alteration (CNA) effects of *PER* (period) and *CRY* (cryptochrome) family members with immune infiltration-related cells, including B cells, cluster of differentiation 4-positive (CD4^+^) T cells, CD8+ T cells, macrophages, neutrophils, and dendritic cells (using TIMER).** (**A**) Correlations of immune infiltration-related cells and expressions of *PER1*, *PER2*, *PER3*, *CRY1*, and *CRY2*. (**B**) CNAs of different circadian factors affect infiltration levels in LUAD. * *p*<0.05, ** *p*<0.01, and *** *p*<0.001, compared to arm-level deletions, the diploid/normal group, and arm-level gain.

In the current research, some relationships between the immune system and circadian rhythms were discovered. For example, it is believed that *PER* and *CRY* are related to the inflammasome [28]. It is, however, unclear whether *PER* and *CRY* are related to immune cell infiltration in LUAD patients. TIMER was utilized to understand relationships between immune infiltration and circadian factor expressions in LUAD (Figure 12A). The analysis showed that expressions of *PER1* and *PER2* were only positively related to infiltration of cluster of differentiation 4-positive (CD4^+^) T cells. *PER3* expression was positively related to the infiltration of B cells, CD8^+^ T cells, CD4^+^ T cells, macrophages, neutrophils, and dendritic cells (DCs). *CRY1* expression was positively related to the infiltration of macrophages and neutrophils. *CRY2* expression was positively related to the infiltration of B cells, CD4^+^ T cells, macrophages, and DCs. These results demonstrated that the circadian-related factors of the *PER* and *CRY* families were related to immune infiltration in LUAD patients.

In addition, somatic copy number alterations (CNAs) of circadian factors were significantly associated with infiltration levels (Figure 12B). Among them, CNAs of *PER1* affected the infiltration level of B cells, CD4^+^ T cells, macrophage, neutrophil, and DCs; CNAs of *PER2* affected the infiltration levels of B cells, CD4^+^ T cells, macrophage, and neutrophils; CNAs of *PER3* affected the infiltration levels of B cells, CD8^+^ T cells, CD4^+^ T cells, and macrophages; CNAs of *CRY1* affected the infiltration levels of B cells, CD4^+^ T cells, and macrophages; and CNAs of *CRY2* affected the infiltration levels of B cells and CD4^+^ T cells. These results demonstrated that genetic alterations of the *PER* and *CRY* families in LUAD led to changes in immune infiltration levels.

**Figure 13:**
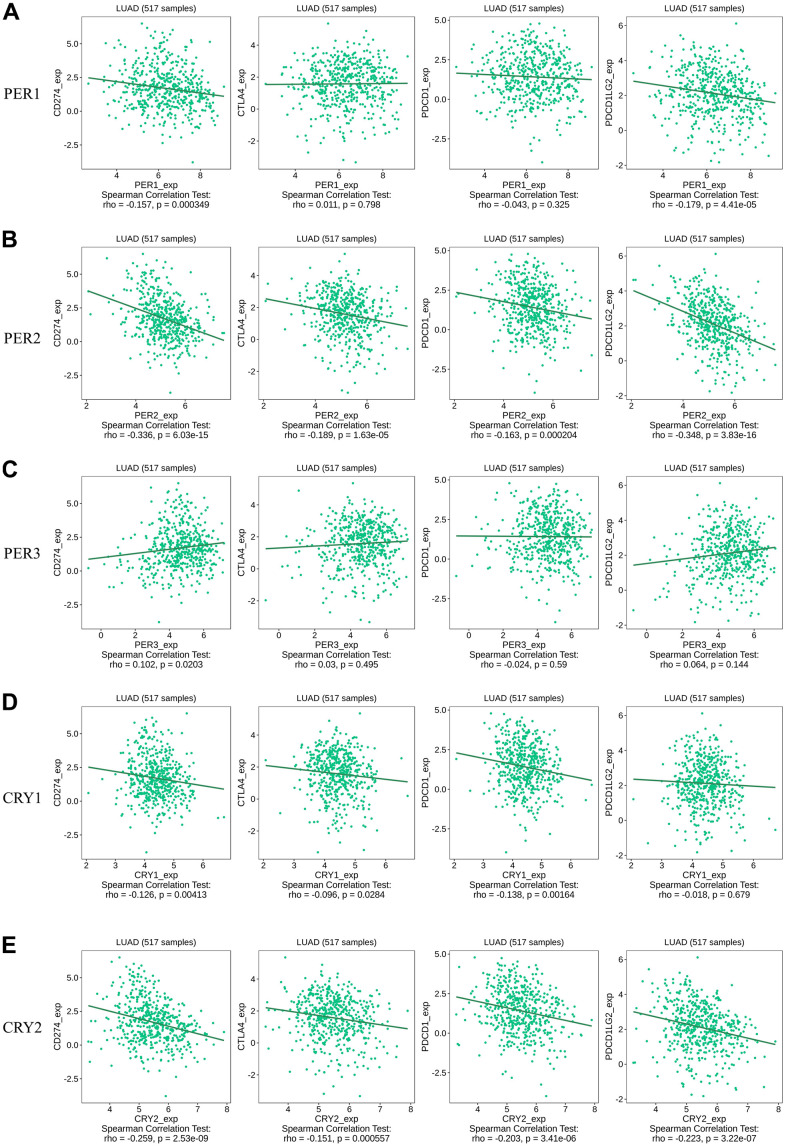
**Spearman correlations between expressions of different circadian factors with *CD274/CTLA4/PDCD1/PDCD1LG2*.** (**A**) Correlations of expressions of immune checkpoint factors *CD274/CTLA4/PDCD1/PDCD1LG2* with *PER1*. (**B**) Correlations of expressions of immune checkpoint factors *CD274/CTLA4/PDCD1/PDCD1LG2* with *PER2*. (**C**) Correlations of expressions of immune checkpoint factors *CD274/CTLA4/PDCD1/PDCD1LG2* with *PER3*. (**D**) Correlations of expressions of immune checkpoint factors *CD274/CTLA4/PDCD1/PDCD1LG2* with *CRY1*. (**E**) Correlations of expressions of immune checkpoint factors *CD274/CTLA4/PDCD1/PDCD1LG2* with *CRY2*.

**Figure 14:**
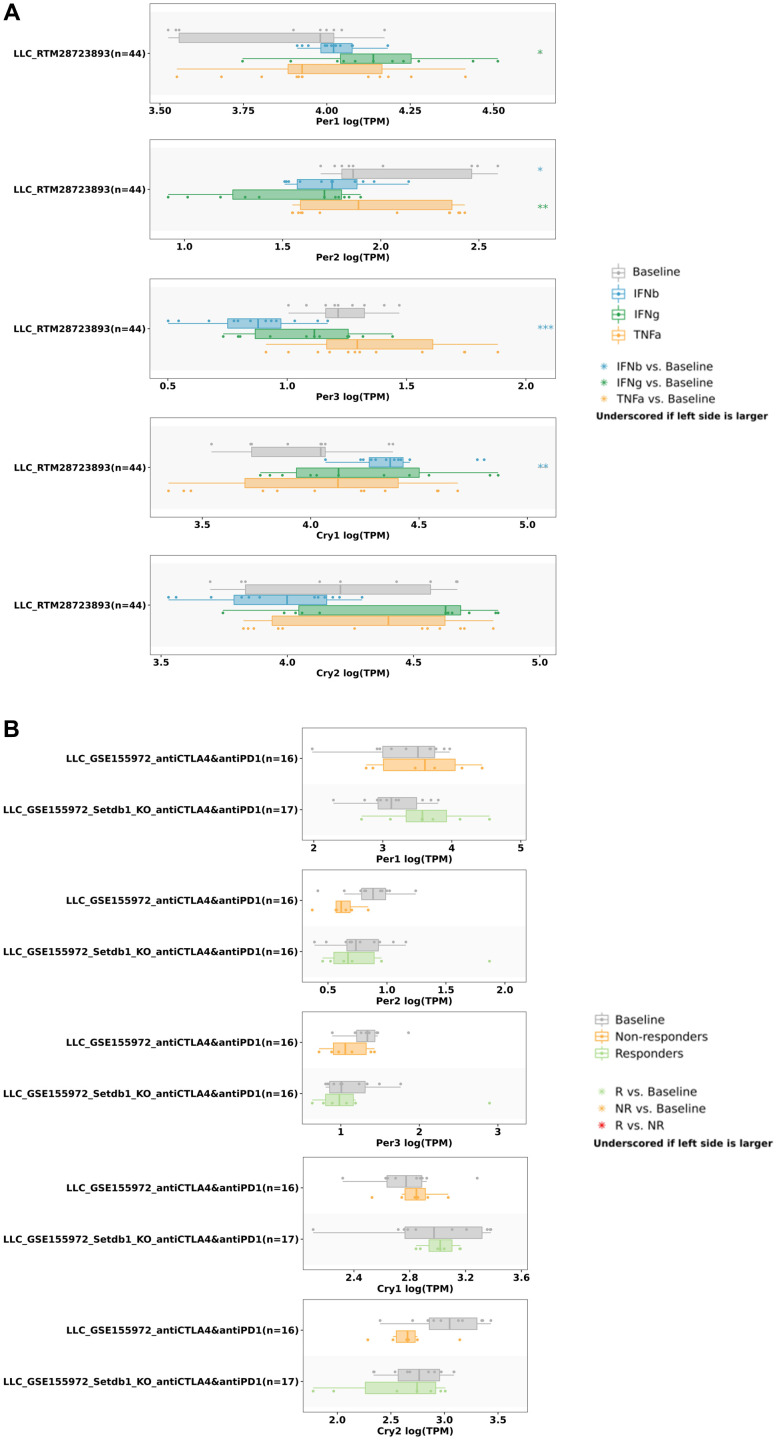
**Correlations of *PER* and *CRY* family members expressions with different cytokine and immune checkpoint blockade treatments.** (**A**) *PER* and *CRY* family member expressions in the LLC lung cancer model stimulated by different cytokines. (**B**) *PER* and *CRY* family member expressions in the LLC lung cancer model were not significantly related to immune checkpoint blockade treatment. * *p*<0.05, ** *p*<0.01, *** *p*<0.001; comparison results are summarized in boxplots.

We also utilized the TISIDB database to understand relationships between various immune checkpoints and circadian factors (Figure 13). The results showed that the expression of *PER1* was correlated with CD274 (rho = -0.157, *p* = 0.000349) and PDCD1LG2 (rho = -0.179, *p* = 4.41E-05) (Figure 13A); the expression of *PER2* was correlated with CD274 (rho = -0.336, *p* = 6.03E-15), CTLA4 (rho = -0.189, *p* = 1.63e-05), PDCD1 (rho = -0.163, *p* = 0.000204), and PDCD1LG2 (rho = -0.348, *p* = 3.83e-16) (Figure 13B); the expression of *PER3* was correlated with CD274 (rho = 0.102, *p* = 0.0203) (Figure 13C); the expression of *CRY1* was correlated with CD274 (rho = -0.126, *p* = 0.00413), CTLA4 (rho = -0.096, *p* = 0.0284), and PDCD1 (rho = -0.138, *p* = 0.00164) (Figure 13D); and the expression of *CRY2* was correlated with CD274 (rho = -0.259, *p* = 2.53E-09), CTLA4 (rho = -0.151, *p* = 0.000557), PDCD1 (rho = -0.203, *p* = 3.41E-06), and PDCD1LG2 (rho = -0.223, p = 3.22e-07) (Figure 13E). Finally, we used the TISMO database to recognize whether the expressions of circadian factors were affected by different immunotherapies (Figure 14). Expressions of circadian rhythm factors in an LLC (lung carcinoma) cancer model changed under stimulation with different cytokines (Figure 14A). However, the expressions of these circadian factors were not significantly affected under different immune checkpoint blockade treatments (Figure 14B).

## DISCUSSION

The physiological behavior of animals often exhibits periodic changes to adapt to repeated environmental changes. The most typical one is the sleep-wake cycle, and others include neurological, metabolic, endocrine, cardiovascular, and immune functions [46]. There are very close links between circadian rhythms and sleep. Both circadian rhythms and sleep play important roles in disease and health, which complicates the process of finding links between circadian rhythms and diseases. This study is the first attempt to elucidate possible links between some gene families associated with circadian rhythms and LUAD.

Previous studies showed that an imbalance of circadian rhythm-related factors may lead to the occurrence of cancer. At present, circadian rhythm-related factors have been discovered, and the basic operation mode of circadian rhythms has been established. *PER* and *CRY* family members were found to be basic factors in modulating circadian rhythms [47], and their relationships with many types of cancer have been explored. Previous studies showed that circadian rhythm-related factors such as the *ARNTL*, *CLOCK*, *RORA*, *RORB*, *CRY1*, *CRY2*, and *PER3* genes were associated with a higher risk of lung cancer [48]. Moreover, some genes of numerous circadian factors can affect the prognosis of NSCLC and changes in immune infiltration and cell functions. Many circadian factors are involved in numerous biological processes, such as inhibiting levels of immune cell infiltration. However, no study has discussed whether the *PER* and *CRY* families have the same effect on lung cancer development [49].

Thus, this is the first study to use bioinformatics to analyze and discuss different *PER* and *CRY* transcription levels, genetic variations, molecular functions, diseases, and their relationships with prognoses and immune infiltration in LUAD patients. We conducted an Oncomine database analysis and found that compared to normal tissues, expressions of circadian rhythm-related factors of *PER1*, *PER2*, *PER3*, *CRY1*, and *CRY2* in LUAD tissues were relatively low. Consistently, expressions of *PER1* and *CRY2* were found in a GEPIA2 analysis to be lower than in normal tissues. Moreover, the KM analysis showed that high expression levels of *PER2*, *PER3*, *CRY1*, and *CRY2* in LUAD patients were related to a better OS.

In addition, *PER* and *CRY* family members were found to have higher mutation rates (29.42%) in LUAD patients. Mutually exclusive coexpressions were found between differentially expressed *PER* and *CRY* family members (mainly coexpression), which meant that LUAD might be induced by the co-inhibition of these gene family members. Furthermore, a molecular mechanism pathway analysis demonstrated that the functions of *PER*- and *CRY*-related genes were mainly involved protein-binding, cell-cycle, histidine-metabolism, and progesterone-mediated oocyte-maturation pathways. In particular, progesterone-mediated oocyte-maturation pathways and the cell cycle were previously demonstrated to be correlated with LUAD [50]. Histidine metabolism was also related to the oncogenic function of *FAM83A* in LUAD [51]. Therefore, the development and inhibition of these pathways may respectively be related to the occurrence and development of LUAD. Results indicated that differentially expressed *PER* and *CRY* family members in LUAD have the potential to become crucial genes for targeted therapy.

We also analyzed MetaCore and found genes that are coexpressed with *PER* and *CRY* family members and the functions associated with those genes. We found that there were many signaling pathways involved in immune evasion, cancer migration and proliferation, and other functions. Among them, *PER1* was related to the Immune response_IL-6 signaling pathway via JAK/STAT. Interleukin (IL)-6 can be found in all human inflammatory diseases and cancers due to its dysregulation and overexpression [52]. It is also known that IL-6-activated Janus kinase 1 (JAK1) might lead to the phosphorylation of Y112 of programmed death ligand 1 (PD-L1) and consequently induce cancer immune evasion [53]. The top three pathways related to *PER2* included a G-protein signaling RhoA regulatory pathway, cell adhesion tight junctions, and development positive regulation of WNT/Beta-catenin signaling in the cytoplasm, which are all related to the function of cell migration [54–56]. Therefore, overexpression of *PER2* may allow cancer cells to easily migrate and spread. Signaling pathways associated with *PER3* were more related to gastrin, including the development of gastrin in cell growth and proliferation, and immune response of gastrin in the inflammatory response. Gastrin is currently considered to be related to cancer development, proliferation, and anti-apoptosis in addition to digestion-related functions [57]. The *CRY1*-related pathway was the WNT signaling pathway. The canonical WNT signaling pathway is associated with cell migration, and the WNT5A non-canonical signaling pathway was also found to be associated with a variety of human cancers [58]. Finally, *CRY2* was mainly related to the cytoskeletal remodeling regulation signaling pathway. The abnormality of cytoskeletal remodeling was related to the invasion and metastasis of cancer cells in previous research [59]. These findings revealed possible related cancer pathways of *PER* and *CRY* family members and provide insights into why dysregulation of circadian rhythms may contribute to cancer development.

Previous studies demonstrated that deregulation of circadian clock genes was indicated in the development of cancers. Melatonin can resynchronize rhythmic patterns of gene expressions, correcting defects in various circadian rhythm oncogenes. Melatonin also inhibits myeloperoxidase catalytic activity [60], which is crucial for tumorigenesis. The action of melatonin requires two receptors known as MT1 and MT2, and these two receptors are present in high densities in the SCN and other organ parts, which may indicate that melatonin affects other organ systems in addition to the SCN [61]. For instance, the mean nocturnal melatonin level ratio and melatonin nocturnal levels decrease in patients with untreated LUAD [62]. Expression levels of the *CRY1* and *BMAL1* core-clock genes were correlated with clinical parameters in epithelial ovarian cancer [63, 64]. Melatonin can inhibit the development of breast cancer by interfering with estrogen [65], and has a certain degree of benefit in colorectal cancer in the elderly [66]. In addition, melatonin has functions of stimulating cell apoptosis, regulating cell survival and tumor-related metabolism, and inhibiting angiogenesis [67]. In the cytokine signaling pathway, PER1 expression is suppressed by tumor necrosis factor (TNF)-α, and knockdown of PER1 decreases the proliferation of pancreatic carcinoma cells [68]. These findings correspond to our results that *PER* and *CRY* family members and related circadian clock genes interfere with melatonin secretion and circadian rhythms, which have effects on pathogeneses of malignancy.

Circadian rhythms can directly interact with components of the immune system, thereby affecting aspects of the immune system such as inflammation. Recent studies also indicated that phagocytosis, migration of inflammatory or infected tissues, cytolytic activity, and proliferative responses to antigens are closely related to circadian rhythms [69]. Furthermore, our data demonstrated that immune cell infiltration in LUAD patients was related to expressions of *PER* and *CRY* family members. In LUAD, expressions of *PER1* and *PER2* were positively correlated with the immune infiltration of cluster of differentiation 4-positive (CD4^+^) T and natural killer (NK) cells. *CRY1* expression was positively correlated with the infiltration of NK cells, macrophages, and neutrophils, and *CRY2* was correlated with the infiltration of NK cells, B cells, CD4^+^ T cells, macrophages, and DCs. *PER3* was connected to the immune infiltration of NK cells, B cells, CD8^+^ T cells, CD4^+^ T cells, macrophages, neutrophils, and DCs. Additionally, it was found that circadian rhythm factors were negatively correlated with the expressions of immune checkpoint-related genes. Further analysis of TISMO found that circadian rhythm factors had no significant relationships with immune checkpoint blockades, but were more related to stimulation of cytokines. These results suggest that circadian rhythm factors may contribute to increases in levels of partial immune infiltration by downregulating expressions of immune checkpoint genes [70–73].

Our results correspond to other research in immunotherapy [74]. *PER1* and *CRY2* were correlated with the expression of CD4^+^ T cells, and the expression of PD-1 exhibited a robust circadian rhythm in normal lung tissues [75], which supported the results that circadian rhythm factors might downregulate expressions of immune checkpoint factors and thus enhance the effects of immunotherapy in LUAD. Although there is a lack of further evident clinical data to prove our hypothesis, we propose a positive association between circadian rhythm factors and immunity.

Taken together, the results indicated that *PER1* and *CRY2* are significantly downregulated in LUAD. Except for *PER1*, high expressions of *PER2*, *PER3*, *CRY1*, and *CRY2* lead to better OS in LUAD patients. In the functional analysis of these circadian coexpressed genes, many factors related to cancer development were also found. In addition, *PER1*, *PER2*, *PER3*, *CRY1*, and *CRY2* were related to six different immune cells to varying degrees, which may be related to the downregulation of different immune checkpoints. Given the above results, these circadian rhythm factors may be involved in tumor immunity of LUAD. *PER* and *CRY* family members could be novel and promising prognostic biomarkers of LUAD.

In summary, we used several high-throughput bioinformatics databases to analyze and investigate gene expressions of *PER* and *CRY* family members and their influences. The present study may help us better understand the molecular functions of circadian rhythm-related factors in LUAD and may provide possible molecular targets for LUAD in chemotherapy and immunotherapy.

## MATERIALS AND METHODS

### UALCAN database analysis

UALCAN (http://ualcan.path.uab.edu/) is an online tool for analyzing cancer OMICS data and was used to analyze the relationship between gene expressions and various cancers. Its functions include (1) analysis of the relative expressions of genes in tumor and normal samples, (2) analysis of the effects of gene expressions on survival rates of different cancer patients, and (3) analysis of high and low expressions of genes in different cancer samples. These helped us understand expression levels of circadian factors in different cancer types [76].

### GeneMANIA

GeneMANIA (https://genemania.org/), an online server for prediction, is used to prioritize genes and predict gene function biological networks [77]. This tool was used to understand relationships between circadian rhythm-related genes in the *PER* and *CRY* families and other genes, and to establish a network.

### Cancer cell line encyclopedia (CCLE) analysis

Cancer cell lines are the most relevant approach in cancer biology research to verify targets and determine drug efficacies (https://portals.broadinstitute.org/ccle). This platform was established with multiple human cancer cell lines (*n* = 1457) and plenty of unique datasets (*n* = 136,488) [78]. Of interest, we obtained gene expression levels in 198 lung cancer cell lines and visualized the data with default settings as in our previous studies [79–82].

### Gene expression profiling interactive analysis dataset analysis 2 (GEPIA2)

GEPIA2 (http://gepia2.cancer-pku.cn/#index), an upgraded version of GEPIA, is a web-based data platform that can be used to compare tumor tissues and normal tissues, and provides 60,498 genes and 198,619 isoforms for querying. Like the older version of GEPIA, functions include differential expression analyses, spectrogram drawing, correlation analyses, patient survival analyses, similar gene detections, and dimensionality reduction analyses. In addition, some of the original older functions have been upgraded, and there are also new functions such as survival maps, isoform use profiling, uploaded expression data comparisons, and cancer-subtype classifiers [83, 84].

### The Kaplan-Meier (KM) plotter analysis

The KM plotter (https://kmplot.com/analysis/), an online database with gene expression and clinical data, can be used to analyze relationships between gene expressions and cancer survival rates. Types of cancer that can be analyzed include lung cancer [85], breast cancer [86], ovarian cancer [87], gastric cancer [88], liver cancer [89] and pan-cancer [90]. We used this tool to understand prognostic values of expression levels of the circadian rhythm-related *PER1, PER2, PER3, CRY1,* and *CRY2* genes in lung cancer patients and analyzed the OS of lung cancer patients under expressions of related genes, as well as the number of patients, median values of messenger (m)RNA expressions, 95% confidence intervals (CIs), hazard ratios (HRs), *p* values, and other related information.

### The cancer genome atlas (TCGA) data and cBioPortal

TCGA is an open database with genome sequencing and related pathological data of more than 30 human tumors [91]. We selected LUAD data (TCGA, PanCancer Atlas) containing 503 pathological reports, and further used the cBioPortal (https://www.cbioportal.org/) to analyze expression levels, coexpressions, and network analyses of circadian rhythm-related genes of *PER* and *CRY* family members [92–94].

### STRING analysis

STRING (https://string-db.org/) is a biological database and web resource for searching and predicting protein-protein interactions (PPIs) [95]. In this study, we used this tool to understand proteins related to the *PER* and *CRY* families, and establish a relationship network.

### DAVID analysis

DAVID (https://david.ncifcrf.gov/) is a database that aims to provide functional explanations for a large number of genes from genome research. DAVID has four analytical modules, namely Annotation Tool, GoCharts, KeggCharts, and DomainCharts [96, 97]. In the study, we used this tool to understand gene functions of the *PER1*, *PER2*, *PER3, CRY1*, and *CRY2* gene lists after cross-comparisons of different databases to evaluate how *PER1, PER2, PER3, CRY1*, and *CRY2* affect molecular functions and may be related to various diseases.

### Tumor immune estimation resource (TIMER) analysis

TIMER (cistrome.shinyapps.io/timer) and its upgraded version TIMER2.0 (http://timer.cistrome.org/) were established to study interactions between malignant cells and host immune systems. It can be used to understand relationships between genes and tumor-infiltrating immune cells and evaluate their clinical impacts [98–101]. This analytical website was used to evaluate the impacts of *PER* and *CRY* family gene expressions on tumor-infiltrating immune cells.

### TISIDB

TISIDB (http://cis.hku.hk/TISIDB/) is an integrated repository portal for tumor-immune system interactions, which integrates multiple heterogeneous data types including the PubMed database, genomics, transcriptomics, and clinical data of 30 cancer types from TCGA, high-throughput screening data, exome and RNA sequencing datasets of patients, and other public databases including UniProt, GO, DrugBank, etc. It can be used to analyze correlations between immune checkpoint factors and circadian factors [102].

### TISMO database analysis

TISMO (http://tismo.cistrome.org/) is a database for hosting and analyzing an extensive collection of syngeneic mouse model data. The entire repository contains raw sequencing data from 1518 mouse samples, including 68 cell lines and 19 cancer types, which can be used to analyze relationships between cancers receiving different treatments (e.g., cytokines and immune checkpoint blockade) and gene expressions of circadian factors [103].

### Functional enrichment analysis

The MetaCore platform was used to identify cancer risk pathways and tumorigenesis in enrichment pathways as we previously described. Expression profiles of TCGA dataset on *PER1*, *PER2*, *PER3*, *CRY1*, and *CRY2* gene expressions were pooled and in-depth integrated to describe potential key candidate genes and pathways in lung cancer [104–107].

### Statistical analysis

We utilized TCGA Pan-Cancer Atlas, a dataset from cBioPortal, to obtain patient data and query the effects of the expressions of different *PER* and *CRY* family members on overall survival (OS). For the survival analysis, a KM plotter was applied, with all default settings, and recurrence-free survival (RFS) was preferred, with the auto-best cutoff values and J best probe set. All possible cutoff values between the lower and upper quartiles were determined, and the best presenting threshold was subsequently used as the cutoff. A log-rank *p* value of <0.05 was considered statistically significant [84, 108, 109].

### Data availability statement

CBioPortal: https://cbioportal.org; The Human Protein Atlas: https://www.proteinatlas.org; Kaplan-Meier plot database https://kmplot.com; MetaCore analysis https://portal.genego.com. The datasets used and/or analyzed during the current study are available from the corresponding author on reasonable request.

## Supplementary Material

Supplementary Figures

Supplementary Table 1

Supplementary Table 2

Supplementary Table 3

Supplementary Table 4

Supplementary Table 5

Supplementary Table 6

Supplementary Table 7
