# Supplementary material for: Circadian rhythm-related factors of PER and CRY family genes function as novel therapeutic targets and prognostic biomarkers in lung adenocarcinoma
Source: Aging (Albany NY). 2022 Nov 16;14(22):9056–89. doi: 10.18632/aging.204386 (PMC9740380; doi:10.18632/aging.204386)
Supplement: Supplementary Figures [file aging-14-204386-s001.pdf]

SUPPLEMENTARY FIGURES

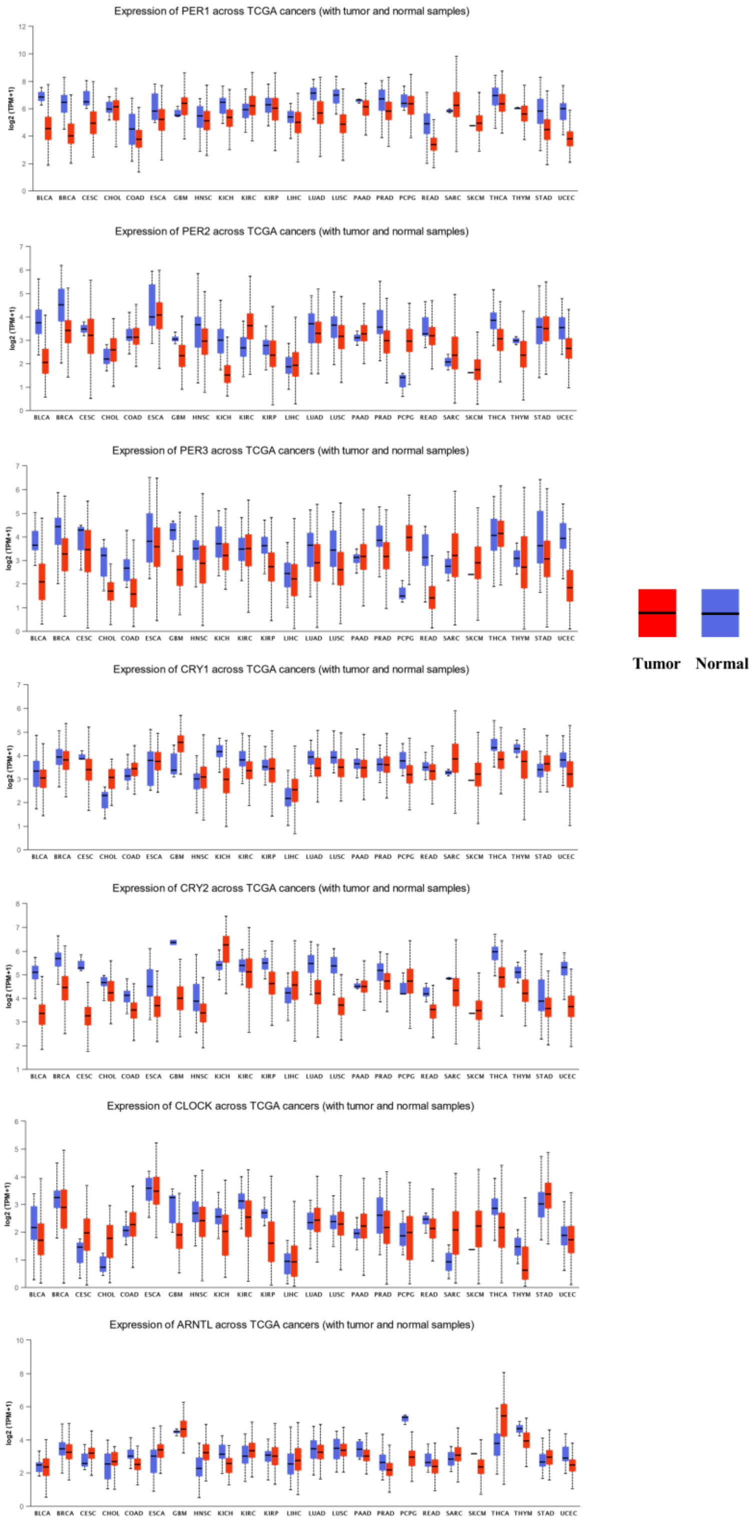

Supplementary Figure 1. Expressions of circadian factor family members in patients with different types of cancer (UALCAN database). The blue box represents the expression of the gene in general tissues, and the red box represents the expression of the gene in cancer.

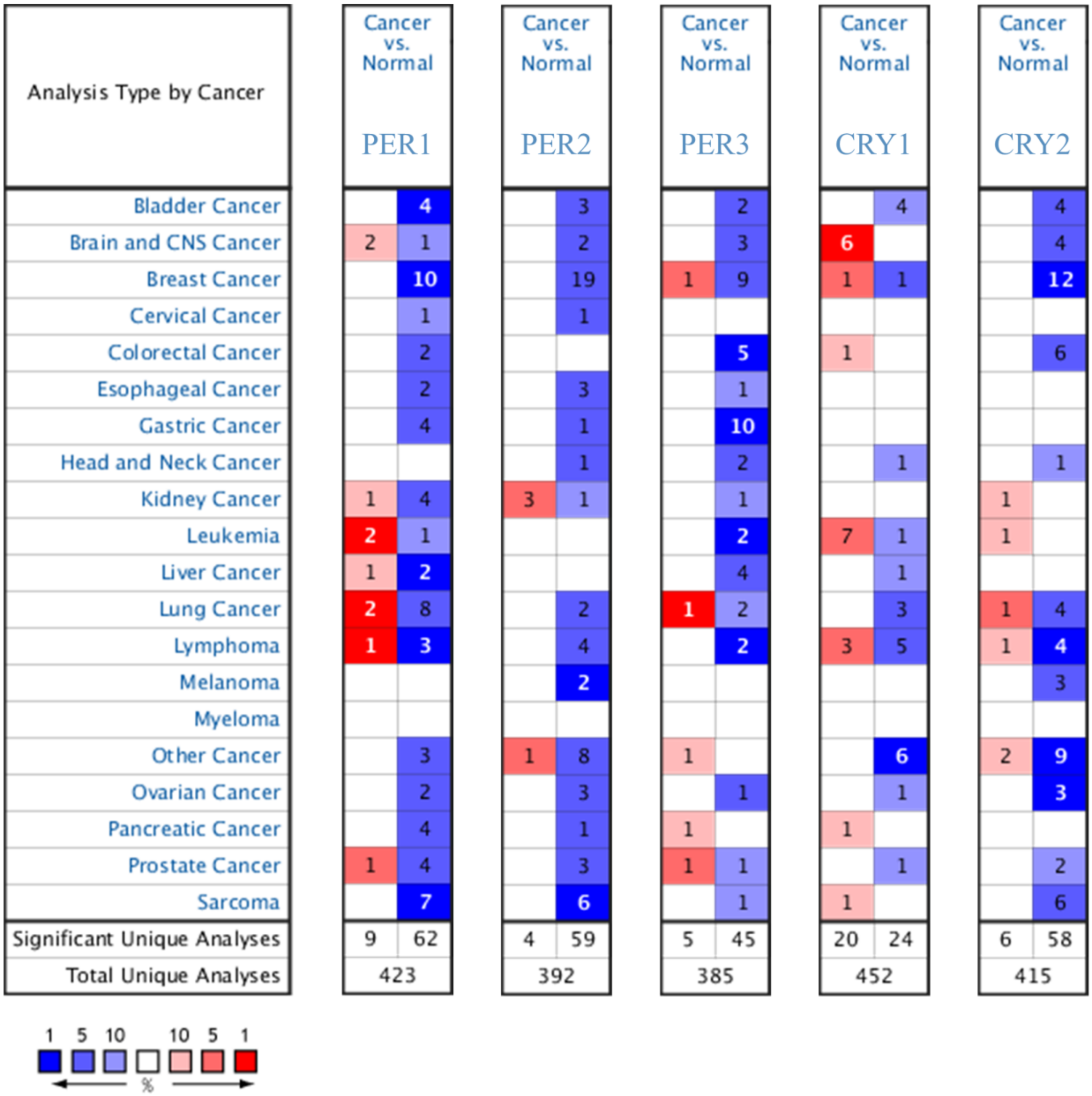

Supplementary Figure 2. Transcription levels of circadian rhythm-related factors of the period (*PER*) and cryptochrome (*CRY*) family members in different types of cancer (Oncomine). This figure shows a dataset with statistically significant mRNA overexpression (red) or downregulated expression (blue) of circadian rhythm-related *PER* and *CRY* family factors with the following parameter design thresholds of a multiple of change of 1.5 and a *p* value of <0.05.

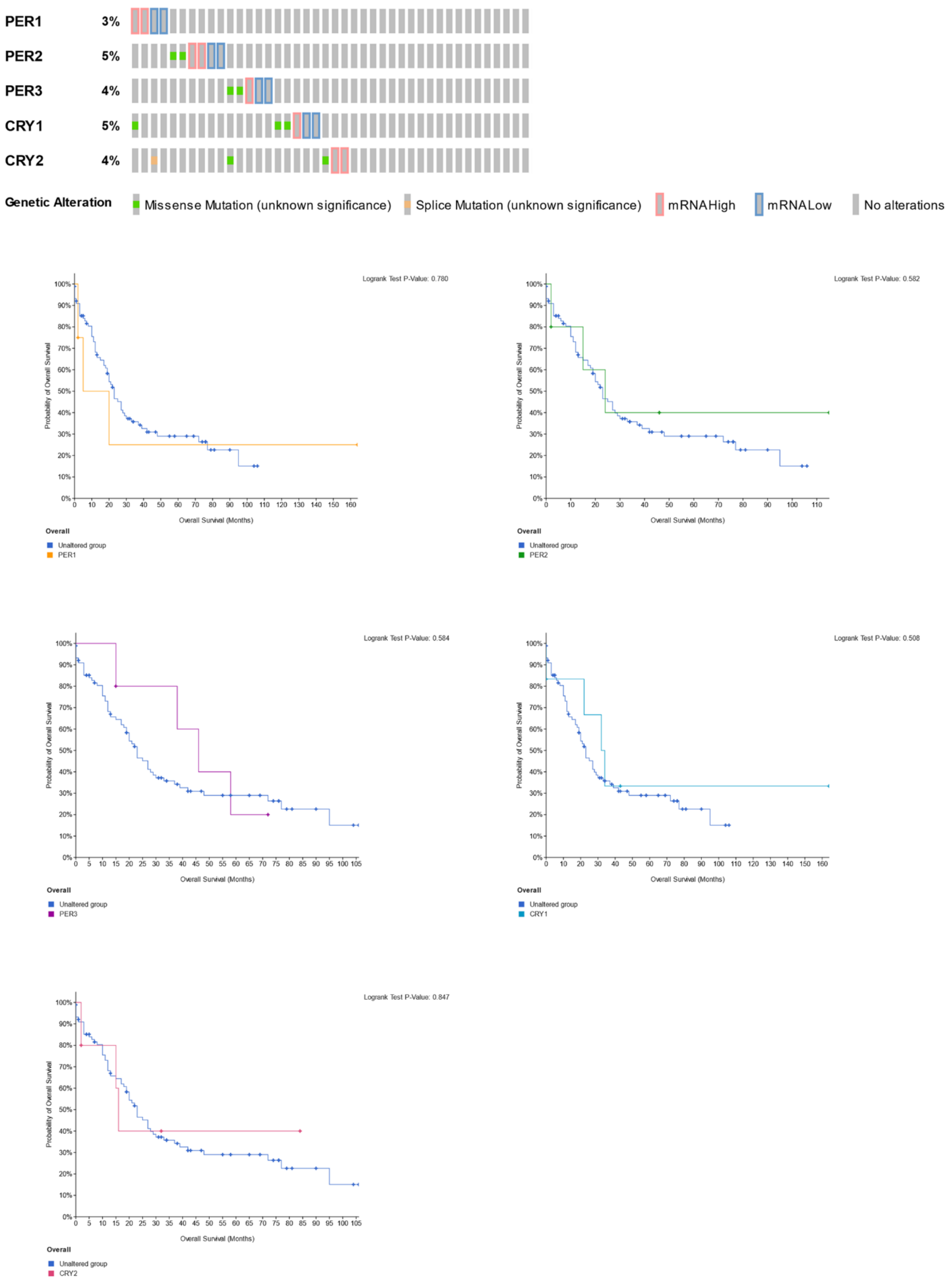

Supplementary Figure 3. cBioPortal analysis of circadian rhythm-related gene changes in small cell lung cancer (SCLC) and its impact on overall survival in SCLC patients.

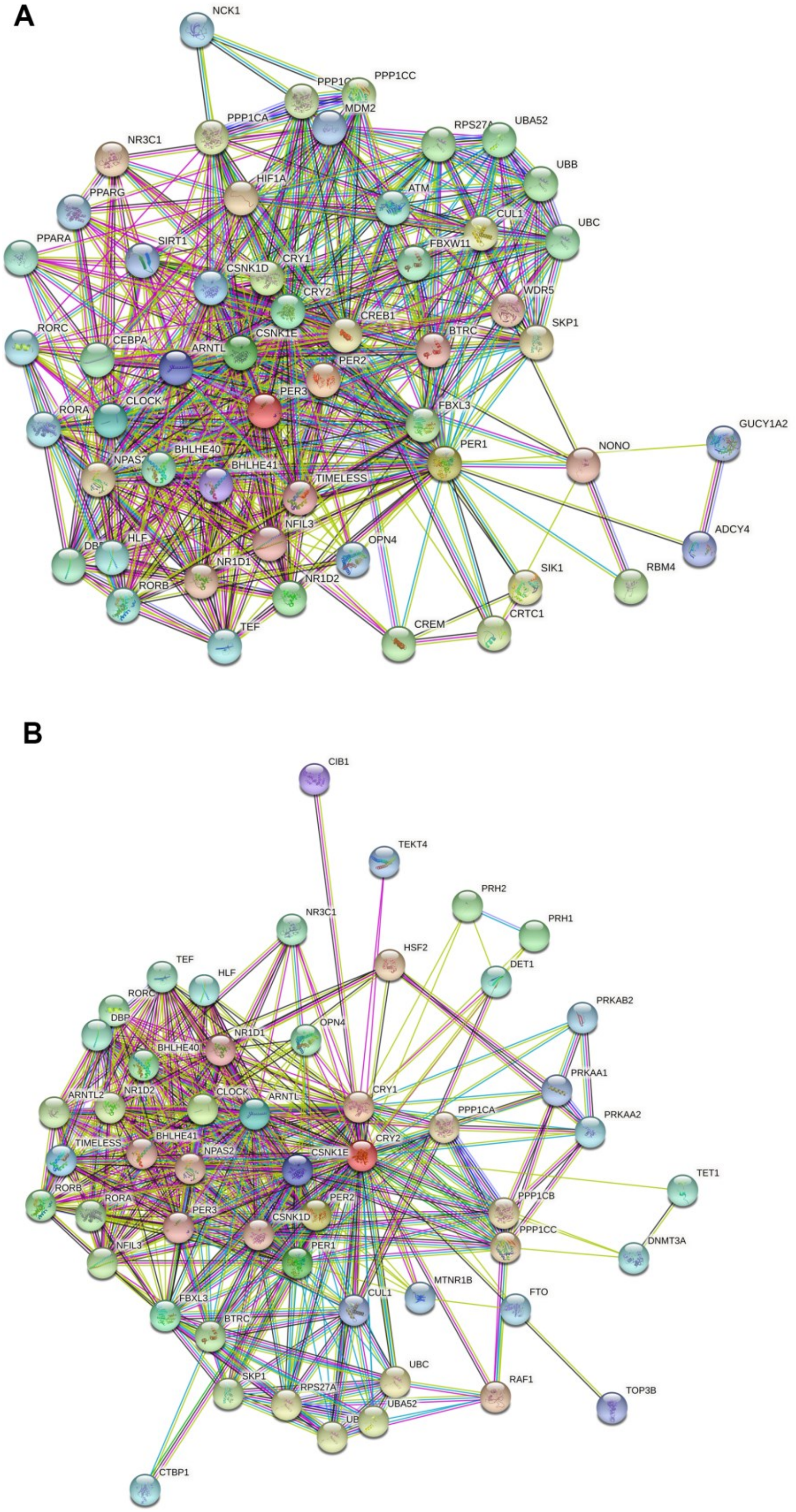

Supplementary Figure 4. Analysis of protein-protein interactions of PER (period) and CRY (cryptochrome) family members of lung adenocarcinoma (LUAD) patients and the network constructed by STRING. (**A**) Network of PER1, PER2, and PER3. (**B**) Network of CRY1 and CRY2.

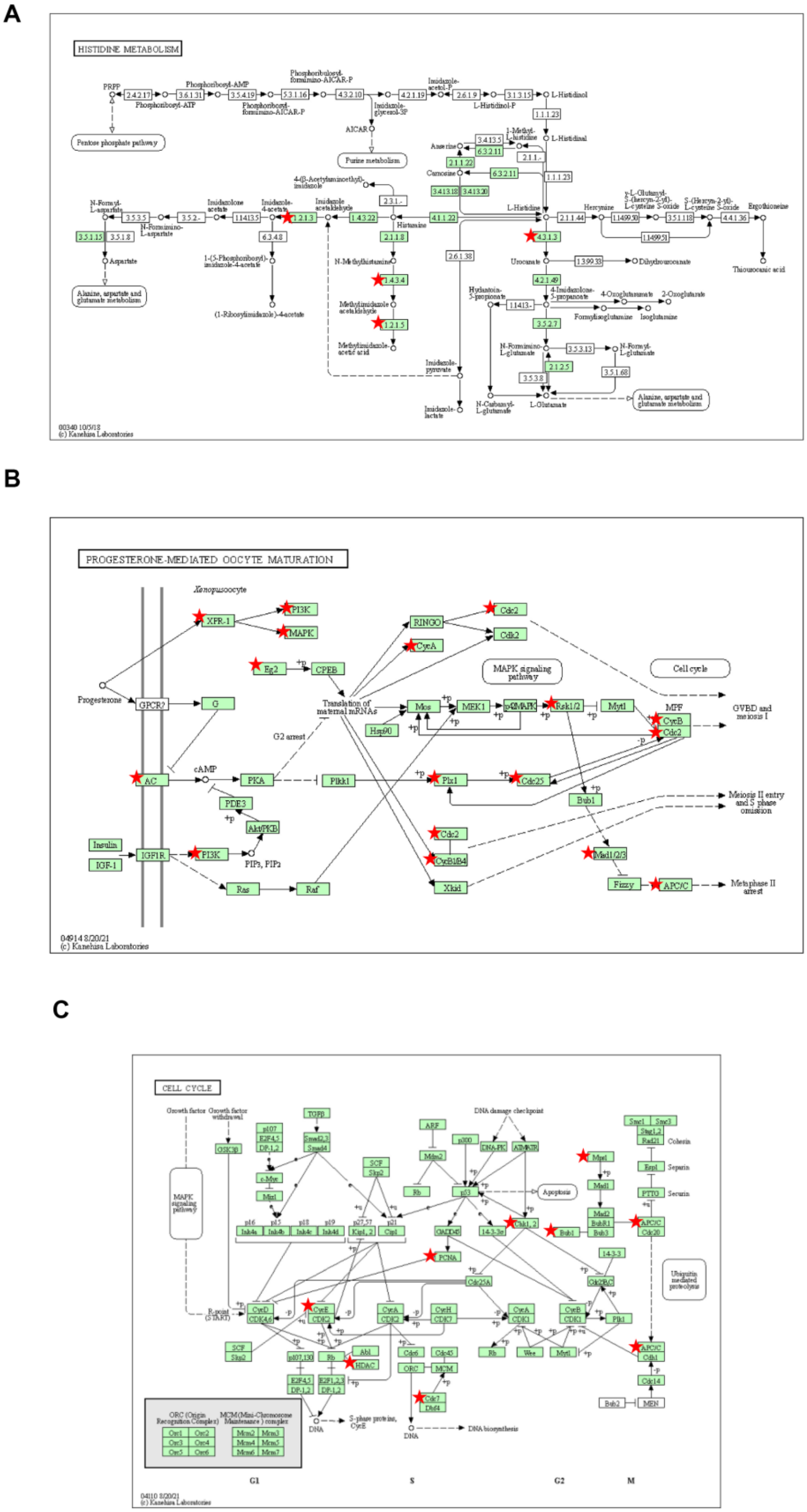

Supplementary Figure 5. KEGG pathway with the highest correlations with individual *PER* (period: *PER1*, *PER2*, and *PER3*), and *CRY* (cryptochrome) family members. (**A**) KEGG pathways of *PER2*. (**B**) KEGG pathways of *PER3*. (**C**) KEGG pathways of *CRY* family members.
