# Supplementary material for: Circadian rhythm-related factors of PER and CRY family genes function as novel therapeutic targets and prognostic biomarkers in lung adenocarcinoma
Source: Aging (Albany NY). 2022 Nov 16;14(22):9056–89. doi: 10.18632/aging.204386 (PMC9740380; doi:10.18632/aging.204386)
Supplement: Supplementary Table 1 [file aging-14-204386-s002.pdf]

SUPPLEMENTARY TABLE

Supplementary Table 1. Significant changes in transcriptional levels of circadian rhythm-related *PER* (period) and *CRY* (cryptochrome) family members in lung adenocarcinoma (LUAD) patients (using the Oncomine database).

	LUAD vs. normal	Fold change	*t*- test	*P*-value	Ref
**PER1**	Lung Adenocarcinoma	-5.555	-5.002	3.35E-5	[1]
	Lung Adenocarcinoma	-1.717	-6.597	1.38E-7	[2]
	Lung Adenocarcinoma	-2.125	-10.850	8.29E-19	[3]
	Lung Adenocarcinoma	-1.861	-5.451	6.23E-7	[4]
	Lung Adenocarcinoma	-2.148	-7.754	1.77E-8	[5]
**PER2**	Lung Adenocarcinoma	-1.803	-5.958	1.29E-7	[4]
**PER3**	Lung Adenocarcinoma	-2.024	-6.727	1.05E-9	[6]
**CRY1**	Lung Adenocarcinoma	-2.058	-5.304	1.39E-6	[4]
	Lung Adenocarcinoma	-1.702	-8.168	5.10E-13	[6]
**CRY2**	Lung Adenocarcinoma	-1.836	-8.556	9.60E-13	[6]
	Lung Adenocarcinoma	-3.450	-4.537	2.47E-5	[4]

REFERENCES

1. Bhattacharjee A, Richards WG, Staunton J, Li C, Monti S, Vasa P, Ladd C, Beheshti J, Bueno R, Gillette M, Loda M, Weber G, Mark EJ, et al. Classification of human lung carcinomas by mRNA expression profiling reveals distinct adenocarcinoma subclasses. Proc Natl Acad Sci U S A. 2001; 98:13790–5. https://doi.org/10.1073/pnas.191502998 PMID:11707567

2. Stearman RS, Dwyer-Nield L, Zerbe L, Blaine SA, Chan Z, Bunn PA Jr, Johnson GL, Hirsch FR, Merrick DT, Franklin WA, Baron AE, Keith RL, Nemenoff RA, et al. Analysis of orthologous gene expression between human pulmonary adenocarcinoma and a carcinogen-induced murine model. Am J Pathol. 2005; 167:1763–75. https://doi.org/10.1016/S0002-9440(10)61257-6 PMID:16314486

3. Landi MT, Dracheva T, Rotunno M, Figueroa JD, Liu H, Dasgupta A, Mann FE, Fukuoka J, Hames M, Bergen AW, Murphy SE, Yang P, Pesatori AC, et al. Gene expression signature of cigarette smoking and its role in lung adenocarcinoma development and survival. PLoS One. 2008; 3:e1651. https://doi.org/10.1371/journal.pone.0001651 PMID:18297132

4. Su LJ, Chang CW, Wu YC, Chen KC, Lin CJ, Liang SC, Lin CH, Whang-Peng J, Hsu SL, Chen CH, Huang CY. Selection of DDX5 as a novel internal control for Q-RT-PCR from microarray data using a block bootstrap re-sampling scheme. BMC Genomics. 2007; 8:140. https://doi.org/10.1186/1471-2164-8-140 PMID:17540040

5. Okayama H, Kohno T, Ishii Y, Shimada Y, Shiraishi K, Iwakawa R, Furuta K, Tsuta K, Shibata T, Yamamoto S, Watanabe S, Sakamoto H, Kumamoto K, et al. Identification of genes upregulated in ALK-positive and EGFR/KRAS/ALK-negative lung adenocarcinomas. Cancer Res. 2012; 72:100–11. https://doi.org/10.1158/0008-5472.CAN-11-1403 PMID:22080568

6. Hou J, Aerts J, den Hamer B, van Ijcken W, den Bakker M, Riegman P, van der Leest C, van der Spek P, Foekens JA, Hoogsteden HC, Grosveld F, Philipsen S. Gene expression-based classification of non-small cell lung carcinomas and survival prediction. PLoS One. 2010; 5:e10312. https://doi.org/10.1371/journal.pone.0010312 PMID:20421987
