# Supplementary material for: Circadian rhythm-related factors of PER and CRY family genes function as novel therapeutic targets and prognostic biomarkers in lung adenocarcinoma
Source: Aging (Albany NY). 2022 Nov 16;14(22):9056–89. doi: 10.18632/aging.204386 (PMC9740380; doi:10.18632/aging.204386)
Supplement: Supplementary Table 2 [file aging-14-204386-s003.docx]

Supplementary Table 2. Analysis of gene oncology (GO) molecular function enrichment and online mendelian inheritance in man (OMIM) diseases of *PER1*, *PER2*, *PER3*, *CRY1,* and *CRY2*, and related genes in lung adenocarcinoma (LUAD) (using the DAVID database).

A. Molecular functions

| Category | Term | Count | % | *p* Value |
| --- | --- | --- | --- | --- |
| GOTERM_MF_DIRECT | protein binding | 389 | 60.2 | 5.4E-13 |
| GOTERM_MF_DIRECT | ATP binding | 92 | 14.2 | 6.3E-8 |
| GOTERM_MF_DIRECT | microtubule binding | 25 | 3.9 | 2.4E-7 |
| GOTERM_MF_DIRECT | single-stranded DNA binding | 16 | 2.5 | 6.6E-7 |
| GOTERM_MF_DIRECT | microtubule motor activity | 13 | 2.0 | 1.8E-5 |
| GOTERM_MF_DIRECT | protein kinase binding | 31 | 4.8 | 2.0E-5 |
| GOTERM_MF_DIRECT | DNA binding | 86 | 13.3 | 1.9E-4 |
| GOTERM_MF_DIRECT | ATP-dependent microtubule motor activity, plus-end-directed | 6 | 0.9 | 2.1E-4 |
| GOTERM_MF_DIRECT | DNA replication origin binding | 5 | 0.8 | 3.9E-4 |
| GOTERM_MF_DIRECT | chromatin binding | 28 | 4.3 | 5.4E-4 |
| GOTERM_MF_DIRECT | 3'-5' DNA helicase activity | 4 | 0.6 | 1.3E-3 |
| GOTERM_MF_DIRECT | protein kinase activity | 25 | 3.9 | 1.7E-3 |
| GOTERM_MF_DIRECT | magnesium ion binding | 17 | 2.6 | 2.0E-3 |
| GOTERM_MF_DIRECT | recombinase activity | 4 | 0.6 | 2.0E-3 |
| GOTERM_MF_DIRECT | single-stranded DNA-dependent ATPase activity | 4 | 0.6 | 4.1E-3 |
| GOTERM_MF_DIRECT | crossover junction endodeoxyribonuclease activity | 4 | 0.6 | 4.1E-3 |
| GOTERM_MF_DIRECT | DNA-dependent ATPase activity | 6 | 0.9 | 4.5E-3 |
| GOTERM_MF_DIRECT | ATPase activity | 15 | 2.3 | 4.6E-3 |
| GOTERM_MF_DIRECT | DNA polymerase binding | 4 | 0.6 | 5.5E-3 |
| GOTERM_MF_DIRECT | glucocorticoid receptor binding | 4 | 0.6 | 5.5E-3 |
| GOTERM_MF_DIRECT | endodeoxyribonuclease activity | 5 | 0.8 | 6.3E-3 |
| GOTERM_MF_DIRECT | pyruvate dehydrogenase (acetyl-transferring) kinase activity | 3 | 0.5 | 6.8E-3 |
| GOTERM_MF_DIRECT | double-stranded DNA binding | 9 | 1.4 | 6.9E-3 |
| GOTERM_MF_DIRECT | transcriptional activator activity, RNA polymerase II core promoter proximal region sequence-specific binding | 17 | 2.6 | 8.2E-3 |
| GOTERM_MF_DIRECT | DNA helicase activity | 5 | 0.8 | 8.7E-3 |
| GOTERM_MF_DIRECT | histone deacetylase binding | 10 | 1.5 | 8.8E-3 |
| GOTERM_MF_DIRECT | four-way junction DNA binding | 4 | 0.6 | 1.1E-2 |
| GOTERM_MF_DIRECT | protein serine/threonine kinase activity | 23 | 3.6 | 1.2E-2 |
| GOTERM_MF_DIRECT | RNA polymerase II core promoter sequence-specific DNA binding | 7 | 1.1 | 1.4E-2 |
| GOTERM_MF_DIRECT | ATP-dependent DNA helicase activity | 5 | 0.8 | 2.4E-2 |
| GOTERM_MF_DIRECT | chemokine activity | 6 | 0.9 | 2.7E-2 |
| GOTERM_MF_DIRECT | identical protein binding | 37 | 5.7 | 2.9E-2 |
| GOTERM_MF_DIRECT | protein homodimerization activity | 36 | 5.6 | 3.3E-2 |
| GOTERM_MF_DIRECT | enzyme binding | 19 | 2.9 | 4.2E-2 |
| GOTERM_MF_DIRECT | nuclease activity | 4 | 0.6 | 4.4E-2 |
| GOTERM_MF_DIRECT | kinase binding | 7 | 1.1 | 4.5E-2 |
| GOTERM_MF_DIRECT | drug binding | 7 | 1.1 | 4.8E-2 |
| GOTERM_MF_DIRECT | gamma-tubulin binding | 4 | 0.6 | 4.9E-2 |

B. OMIM_DISEASES

| Category | Term | Count | % | *p* Value |
| --- | --- | --- | --- | --- |
| OMIM_DISEASE | Breast cancer, susceptibility to | 5 | 0.8 | 4.9E-5 |
| OMIM_DISEASE | Colorectal cancer, somatic | 4 | 0.6 | 1.1E-2 |

C. Molecular functions

| Category | Term | Count | % | *p* Value |
| --- | --- | --- | --- | --- |
| GOTERM_MF_DIRECT | protein binding | 371 | 58.9 | 1.3E-12 |
| GOTERM_MF_DIRECT | poly(A) RNA binding | 59 | 9.4 | 5.0E-4 |
| GOTERM_MF_DIRECT | single-stranded DNA binding | 11 | 1.7 | 9.6E-4 |
| GOTERM_MF_DIRECT | microtubule binding | 16 | 2.5 | 3.8E-3 |
| GOTERM_MF_DIRECT | chromatin binding | 24 | 3.8 | 5.4E-3 |
| GOTERM_MF_DIRECT | guanyl-nucleotide exchange factor activity | 11 | 1.7 | 5.6E-3 |
| GOTERM_MF_DIRECT | ubiquitin conjugating enzyme activity | 5 | 0.8 | 1.4E-2 |
| GOTERM_MF_DIRECT | CCR1 chemokine receptor binding | 3 | 0.5 | 2.0E-2 |
| GOTERM_MF_DIRECT | chemokine activity | 6 | 1.0 | 2.2E-2 |
| GOTERM_MF_DIRECT | protein kinase binding | 21 | 3.3 | 2.4E-2 |
| GOTERM_MF_DIRECT | patched binding | 3 | 0.5 | 2.7E-2 |
| GOTERM_MF_DIRECT | CCR5 chemokine receptor binding | 3 | 0.5 | 2.7E-2 |
| GOTERM_MF_DIRECT | ubiquitin-like protein transferase activity | 3 | 0.5 | 2.7E-2 |
| GOTERM_MF_DIRECT | ubiquitin protein ligase binding | 17 | 2.7 | 2.8E-2 |
| GOTERM_MF_DIRECT | structural constituent of cytoskeleton | 9 | 1.4 | 2.9E-2 |
| GOTERM_MF_DIRECT | actin filament binding | 10 | 1.6 | 3.1E-2 |
| GOTERM_MF_DIRECT | ribonuclease activity | 4 | 0.6 | 3.9E-2 |
| GOTERM_MF_DIRECT | GDP-dissociation inhibitor activity | 3 | 0.5 | 4.1E-2 |
| GOTERM_MF_DIRECT | Rho guanyl-nucleotide exchange factor activity | 7 | 1.1 | 4.1E-2 |
| GOTERM_MF_DIRECT | 14-3-3 protein binding | 4 | 0.6 | 4.3E-2 |
| GOTERM_MF_DIRECT | glucocorticoid receptor binding | 3 | 0.5 | 4.9E-2 |
| GOTERM_MF_DIRECT | IgG binding | 3 | 0.5 | 4.9E-2 |

D. OMIM_DISEASES

| Category | Term | Count | % | *p* Value |
| --- | --- | --- | --- | --- |
| OMIM_DISEASE | Skin/hair/eye pigmentation 1, blue/nonblue eyes | 2 | 0.3 | 4.8E-2 |
| OMIM_DISEASE | Skin/hair/eye pigmentation 1, blond/brown hair | 2 | 0.3 | 4.8E-2 |

E. Molecular functions

| Category | Term | Count | % | *p* Value |
| --- | --- | --- | --- | --- |
| GOTERM_MF_DIRECT | protein binding | 416 | 58.3 | 3.3E-11 |
| GOTERM_MF_DIRECT | poly(A) RNA binding | 84 | 11.8 | 4.5E-9 |
| GOTERM_MF_DIRECT | MHC class II receptor activity | 6 | 0.8 | 1.7E-4 |
| GOTERM_MF_DIRECT | metal ion binding | 108 | 15.1 | 5.2E-4 |
| GOTERM_MF_DIRECT | structural constituent of ribosome | 20 | 2.8 | 8.5E-4 |
| GOTERM_MF_DIRECT | protein kinase binding | 28 | 3.9 | 1.2E-3 |
| GOTERM_MF_DIRECT | core promoter sequence-specific DNA binding | 7 | 1.0 | 5.4E-3 |
| GOTERM_MF_DIRECT | DNA replication origin binding | 4 | 0.6 | 7.2E-3 |
| GOTERM_MF_DIRECT | cadherin binding involved in cell-cell adhesion | 21 | 2.9 | 7.7E-3 |
| GOTERM_MF_DIRECT | single-stranded DNA binding | 10 | 1.4 | 8.8E-3 |
| GOTERM_MF_DIRECT | alpha-amylase activity | 3 | 0.4 | 1.3E-2 |
| GOTERM_MF_DIRECT | protein binding involved in protein folding | 4 | 0.6 | 1.5E-2 |
| GOTERM_MF_DIRECT | integrin binding | 10 | 1.4 | 1.9E-2 |
| GOTERM_MF_DIRECT | porin activity | 3 | 0.4 | 2.0E-2 |
| GOTERM_MF_DIRECT | miRNA binding | 4 | 0.6 | 2.1E-2 |
| GOTERM_MF_DIRECT | MHC class II protein complex binding | 4 | 0.6 | 2.1E-2 |
| GOTERM_MF_DIRECT | protein serine/threonine/tyrosine kinase activity | 5 | 0.7 | 2.3E-2 |
| GOTERM_MF_DIRECT | protein kinase activity | 22 | 3.1 | 3.5E-2 |
| GOTERM_MF_DIRECT | protein transmembrane transporter activity | 3 | 0.4 | 3.5E-2 |
| GOTERM_MF_DIRECT | phosphatidylinositol 3-kinase regulatory subunit binding | 3 | 0.4 | 4.4E-2 |
| GOTERM_MF_DIRECT | RNA binding | 30 | 4.2 | 4.6E-2 |
| GOTERM_MF_DIRECT | ATPase activity | 13 | 1.8 | 4.7E-2 |
| GOTERM_MF_DIRECT | ubiquitin protein ligase binding | 18 | 2.5 | 4.8E-2 |
| GOTERM_MF_DIRECT | MAP kinase kinase kinase activity | 4 | 0.6 | 4.9E-2 |

F. OMIM_DISEASES

| Category | Term | Count | % | *p* Value |
| --- | --- | --- | --- | --- |
| OMIM_DISEASE | Afibrinogenemia, congenital | 3 | 0.4 | 3.0E-3 |
| OMIM_DISEASE | Dysfibrinogenemia, congenital | 3 | 0.4 | 3.0E-3 |

G. Molecular functions

| Category | Term | Count | % | *p* Value |
| --- | --- | --- | --- | --- |
| GOTERM_MF_DIRECT | protein binding | 62 | 75.6 | 2.9E-7 |
| GOTERM_MF_DIRECT | single-stranded DNA-dependent ATPase activity | 4 | 4.9 | 1.0E-5 |
| GOTERM_MF_DIRECT | protein kinase binding | 9 | 11.0 | 2.8E-4 |
| GOTERM_MF_DIRECT | DNA clamp loader activity | 3 | 3.7 | 5.5E-4 |
| GOTERM_MF_DIRECT | ATP binding | 16 | 19.5 | 2.4E-3 |
| GOTERM_MF_DIRECT | damaged DNA binding | 4 | 4.9 | 2.9E-3 |
| GOTERM_MF_DIRECT | DNA helicase activity | 3 | 3.7 | 5.2E-3 |
| GOTERM_MF_DIRECT | gamma-tubulin binding | 3 | 3.7 | 5.2E-3 |
| GOTERM_MF_DIRECT | protein serine/threonine kinase activity | 7 | 8.5 | 6.9E-3 |
| GOTERM_MF_DIRECT | ubiquitin protein ligase binding | 6 | 7.3 | 9.5E-3 |
| GOTERM_MF_DIRECT | dinucleotide insertion or deletion binding | 2 | 2.4 | 1.3E-2 |
| GOTERM_MF_DIRECT | protein homodimerization activity | 9 | 11.0 | 1.7E-2 |
| GOTERM_MF_DIRECT | uracil DNA N-glycosylase activity | 2 | 2.4 | 2.2E-2 |
| GOTERM_MF_DIRECT | protein kinase activity | 6 | 7.3 | 2.3E-2 |
| GOTERM_MF_DIRECT | MutLalpha complex binding | 2 | 2.4 | 2.7E-2 |
| GOTERM_MF_DIRECT | mismatched DNA binding | 2 | 2.4 | 4.8E-2 |

(A) Analysis of GO molecular functions of *PER1*. (B) Analysis of OMIM diseases of *PER1*. (C) Analysis of GO molecular functions of *PER2*. (D) Analysis of OMIM diseases of *PER2*. (E) Analysis of GO molecular functions of *PER3*. (F) Analysis of OMIM diseases of *PER3*. (G) Analysis of GO molecular functions of *CRY1* and *CRY2*.
