# Supplementary material for: Circadian rhythm-related factors of PER and CRY family genes function as novel therapeutic targets and prognostic biomarkers in lung adenocarcinoma
Source: Aging (Albany NY). 2022 Nov 16;14(22):9056–89. doi: 10.18632/aging.204386 (PMC9740380; doi:10.18632/aging.204386)
Supplement: Supplementary Table 3 [file aging-14-204386-s004.docx]

Supplementary Table 3. Pathway analysis of genes coexpressed with *PER1* from public lung cancer databases using the MetaCore database (with *p*<0.05 set as the cutoff value).

| No. | Map | *p* Value | Network objects from active data |
| --- | --- | --- | --- |
| 1 | Signal transduction_Beta-adrenergic receptors signaling via Cyclic AMP | 1.018E-06 | CACNB2, KCNQ1, Phospholemman, NOR1, Beta-2 adrenergic receptor, NURR1,  Beta-1 adrenergic receptor, NUR77, RRAD, Adenylate cyclase |
| 2 | Immune response_IL-6 signaling pathway via JAK/STAT | 2.218E-06 | c-Jun/c-Fos, SOCS2, AP-1, CISH, c-Fos, sIL6-RA, FKHR, Osteocalcin,  C/EBPbeta, Mcl-1, c-Jun, IL6RA, C/EBPdelta |
| 3 | Signal transduction_Calcium-mediated signaling | 2.614E-06 | I-kB, PPARGC1 (PGC1-alpha), Myocardin, NURR1, PKC, HDAC5,  NUR77, c-Fos, MYH11, NF-AT2(NFATC1), CaMKK, MUNC13, c-Jun |
| 4 | Development_Beta adrenergic receptors in brown adipocyte differentiation | 5.682E-06 | PPARGC1 (PGC1-alpha), Beta-2 adrenergic receptor,  Beta-1 adrenergic receptor, C/EBPbeta, KLF4, Adenylate cyclase,  Factor D, LPL, C/EBPdelta |
| 5 | Development_Oligodendrocyte differentiation from adult stem cells | 1.768E-05 | PLP1, MCT8, CNTN1 (F3), EDNRB, SMAD6, PTCH1, CNTN6,  Endothelin-1, BMP4, BMP2 |
| 6 | Production and activation of TGF-beta in airway smooth muscle cells | 3.848E-05 | AGTR1, Leukocyte elastase, c-Jun/c-Fos, AP-1,  TGF-beta receptor type III (betaglycan), c-Fos, NFKBIA, c-Jun |
| 7 | Apoptosis and survival_IL-17-induced CIKS-independent signaling pathways | 4.745E-05 | TPL2(MAP3K8), c-Jun/c-Fos, AP-1, IL-17RC, c-Fos,  C/EBPbeta, c-Jun, MKP-1, C/EBPdelta |
| 8 | Immune response_IL-6-induced acute-phase response in hepatocytes | 4.785E-05 | c-Jun/c-Fos, c-Fos, FKHR, A2M, C/EBPbeta, c-Jun, IL6RA, C/EBPdelta |
| 9 | Skeletal muscle atrophy in COPD | 4.872E-05 | Desmin, Beta TnTF, I-kB, Tuberin, PPARGC1 (PGC1-alpha),  MuRF1, JunD, FKHR, MyHC, NFKBIA |
| 10 | Development_Neural stem cell lineage commitment (schema) | 7.233E-05 | PLP1, CNTN1 (F3), PTCH1, BAG-1, CNTN6, ATP1A2, BMP4, BMP2 |
| 11 | Glucocorticoids-mediated inhibition of pro-constrictory and pro-inflammatory signaling in airway smooth muscle cells | 7.998E-05 | Beta-2 adrenergic receptor, CPI-17, c-Fos, PDE4D, PLA2, NFKBIA, MRLC, c-Jun, MKP-1 |
| 12 | Development_PTHR1 in bone and cartilage development | 1.627E-04 | PTHR1, c-Jun/c-Fos, NURR1, Ihh, PKC, c-Fos, PTCH1,  Osteocalcin, Adenylate cyclase, BMP2, MKP-1 |
| 13 | Immune response_IL-3 signaling via JAK/STAT, p38, JNK and NF-kB | 1.970E-04 | ID1, I-kB, NOTCH4, CISH, Cyclin D3, IRE1, c-Fos,  Bcl-6, C/EBPbeta, Mcl-1, BMP2, MKP-1 |
| 14 | Immune response_IL-5 signaling via JAK/STAT | 2.331E-04 | SOCS2, CISH, Cyclin D3, c-Fos, Bcl-6, Mcl-1, NFKBIA, c-Jun, MKP-1 |
| 15 | Immune response_IL-11 signaling via JAK/STAT | 2.385E-04 | IL11RA, Leukocyte elastase, c-Fos, A2M, sIL11-RA, Pim-3, SP-B |
| 16 | Retinal ganglion cell damage in glaucoma | 2.526E-04 | Factor H, TBC1D17, EDNRB, Endothelin-1, A2M, TrkC, c-Jun, NSGPeroxidase |
| 17 | Development_Stimulation of differentiation of mouse embryonic fibroblasts into adipocytes by extracellular factors | 3.242E-04 | Tuberin, IRS-2, FKHR, C/EBPbeta, KLF15,  Adenylate cyclase, Factor D, BMP2, LPL, C/EBPdelta |
| 18 | Reproduction_Gonadotropin-releasing hormone (GnRH) signaling | 4.077E-04 | c-Jun/c-Fos, AP-1, HDAC5, NUR77, c-Fos, ATF-3,  Adenylate cyclase, c-Jun, MKP-1, FosB |
| 19 | Blood coagulation_Blood coagulation | 5.788E-04 | Coagulation factor X, Thrombomodulin, CPB2, Tissue factor,  A2M, SERPINF2, Coagulation factor XI |
| 20 | Cooperative action of IFN-gamma and TNF-alpha on astrocytes in multiple sclerosis | 5.788E-04 | I-kB, Beta-2 adrenergic receptor, IP10, C/EBPbeta,  Adenylate cyclase, NFKBIA, C/EBPdelta |
| 21 | Histone deacetylases in Prostate Cancer | 6.510E-04 | HDAC10, Sirtuin3, HDAC6, HDAC5, HSP90, FKHR |
| 22 | Signal transduction_Angiotensin II/ AGTR1 signaling via RhoA and JNK | 6.976E-04 | AGTR1, c-Jun/c-Fos, AP-1, CTGF, c-Fos, EPHX2,  Endothelin-1, MRLC, c-Jun, ARHGEF1 (p115RhoGEF) |
| 23 | Muscle contraction_Regulation of eNOS activity in endothelial cells | 7.275E-04 | EPAS1, ETS2, AP-1, KLF2, EDNRB, HSP90, Endothelin-1, c-Jun, FosB |
| 24 | NF-AT signaling in cardiac hypertrophy | 7.275E-04 | AGTR1, PRKD1, ERK5 (MAPK7), HDAC5,  Beta-1 adrenergic receptor, Endothelin-1, Troponin T, cardiac, CaMKK, CAMTA2 |
| 25 | Development_Insulin, IGF-1 and TNF-alpha in brown adipocyte differentiation | 7.965E-04 | PPARGC1 (PGC1-alpha), Beta-2 adrenergic receptor,  Beta-1 adrenergic receptor, IRS-2, FKHR, C/EBPbeta, Factor D, C/EBPdelta |
| 26 | Immune response_IL-4 signaling pathway | 8.381E-04 | PKC-zeta, Tuberin, AP-1, c-Fes, PKC, JunD, IRS-2, FKHR, PDE4, NFKBIA, c-Jun |
| 27 | Putative glucocorticoid- and LABA-mediated inhibition of pro-fibrotic signaling in airway fibroblasts/myofibroblasts | 9.463E-04 | c-Jun/c-Fos, AP-1, Beta-2 adrenergic receptor, cAMP-GEFI, c-Jun, MKP-1 |
| 28 | Signal transduction_mTORC2 downstream signaling | 1.015E-03 | PKC-zeta, Tuberin, SGK1, OSR1, PKC, STK4, FKHR, Mcl-1, NEDD4L |
| 29 | Regulation of Tissue factor signaling in cancer | 1.064E-03 | EPAS1, JunD/c-Fos, AP-1, JunD, Tissue factor, c-Jun/c-Jun, c-Jun |
| 30 | Transcription_Negative regulation of HIF1A function | 1.128E-03 | KLF2, HIF3A, HIF-prolyl hydroxylase, EGLN2,  FHL1 (SLIM1), Sirtuin3, HSP90, CITED2, EAF2 |
| 31 | Signal transduction_PDGF signaling via PI3K/AKT and NFkB pathways | 1.252E-03 | ETS2, SGK1, Thrombomodulin, Myocardin,  c-Fos, MYH11, FKHR, NFKBIA, c-Jun |
| 32 | Role of GSK3 beta in cardioprotection against myocardial infarction | 1.335E-03 | Epo receptor, Metenkefalin, RAMP2, RAMP3, ANT, Enkephalin A |
| 33 | Development_Schema: SMAD-dependent TGF-beta family signaling in embryonic stem cells | 1.796E-03 | ID1, Lefty-2, BMP4, SOX17, BMP2 |
| 34 | Immune response_Histamine H1 receptor signaling in immune response | 1.823E-03 | I-kB, c-Jun/c-Fos, c-Fos, Tissue factor, NF-AT2(NFATC1), NFKBIA, c-Jun |
| 35 | Inhibition of remyelination in multiple sclerosis: role of cell-cell and ECM-cell interactions | 1.823E-03 | PLP1, HYAL2, PKC, HYAL1, CD81, Connexin 47, Neurofascin |
| 36 | Immune response_IL-17 signaling pathways | 1.825E-03 | I-kB, c-Jun/c-Fos, IL-17RC, c-Fos, G-CSF, C/EBPbeta, c-Jun, C/EBPdelta |
| 37 | Development_Ligand-dependent activation of the ESR1/AP-1 pathway | 1.998E-03 | c-Jun/c-Fos, AP-1, c-Fos, c-Jun |
| 38 | Muscle contraction_Relaxin signaling pathway | 2.066E-03 | PKC-zeta, I-kB, PDE8B, EDNRB, PDE4D, Endothelin-1, NFKBIA |
| 39 | Neurogenesis_NGF/ TrkA MAPK-mediated signaling | 2.084E-03 | PKC-zeta, SGK1, ERK5 (MAPK7), AP-1, NUR77, c-Fos,  JMJD3, Ephrin-A receptor 2, SH2B, c-Jun, FosB |
| 40 | Fibroblast/ myofibroblast proliferation in asthmatic airways | 2.466E-03 | EDNRB, CTGF, PKC, Tissue factor, Endothelin-1, c-Jun |
| 41 | Muscle contraction_Delta-type opioid receptor in smooth muscle contraction | 2.611E-03 | Metenkefalin, CPI-17, Leu-enkephalin, MRLC, PLC-delta 1 |
| 42 | Proteases and EGFR-activated mucin production in airway epithelium in COPD | 2.626E-03 | Leukocyte elastase, PPARGC1 (PGC1-alpha), c-Jun/c-Fos,  AP-1, DUOX1, NFKBIA, c-Jun |
| 43 | TNF-alpha-induced inflammatory signaling in normal and asthmatic airway epithelium | 2.836E-03 | I-kB, IP10, c-Fos, TWEAK(TNFSF12), NFKBIA, c-Jun |
| 44 | Role of Endothelin-1 in inflammation and vasoconstriction in Sickle cell disease | 2.836E-03 | c-Jun/c-Fos, AP-1, EDNRB, c-Fos, Endothelin-1, c-Jun |
| 45 | WNT signaling in proliferative-type melanoma cells | 2.836E-03 | Axin2, NURR1, NUR77, Dsh, WIF1, Axin |
| 46 | HDL dyslipidemia in type 2 diabetes and metabolic syndrome X | 2.836E-03 | Pre beta-1 HDL, S1P1 receptor, APOA1, HDL, Nascent HDL, LCAT |
| 47 | Histamine metabolism | 3.106E-03 | ALDH2, MAOA, AOC3, HDC, MAOB |
| 48 | Chemotaxis_Lysophosphatidic acid signaling via GPCRs | 3.627E-03 | PKC-zeta, Rho GTPase, PRKD1, AP-1, CTGF,  PKC, c-Fos, FKHR, PLC-delta 1, c-Jun, E3KARP (NHERF2), ARHGEF1 (p115RhoGEF) |
| 49 | ENaC regulation in normal and CF airways | 3.677E-03 | gamma-ENaC, EDNRB, Beta-2 adrenergic receptor, cAMP-GEFI,  Endothelin-1, Adenylate cyclase, NEDD4L |
| 50 | IL-6 signaling in breast cancer cells | 3.677E-03 | MUC1, IP10, c-Fos, C/EBPbeta, Mcl-1, IL6RA, C/EBPdelta |
