# Supplementary material for: Circadian rhythm-related factors of PER and CRY family genes function as novel therapeutic targets and prognostic biomarkers in lung adenocarcinoma
Source: Aging (Albany NY). 2022 Nov 16;14(22):9056–89. doi: 10.18632/aging.204386 (PMC9740380; doi:10.18632/aging.204386)
Supplement: Supplementary Table 4 [file aging-14-204386-s005.docx]

Supplementary Table 4. Pathway analysis of genes coexpressed with *PER2* from public lung cancer databases using the MetaCore database (with *p*<0.05 set as the cutoff value).

| No. | Map | *p* Value | Network objects from active data |
| --- | --- | --- | --- |
| 1 | G-protein signaling_RhoA regulation pathway | 5.859E-08 | p200RhoGAP, IGF-1 receptor, ROCK, Fyn, DBS, Ephrin-A receptors,  GRAF, G-protein alpha-q/11, VIL2 (ezrin) |
| 2 | Cell adhesion_Tight junctions | 6.447E-07 | JAM1, INADL, ROCK, ZO-3, Cingulin, PARD3, ZO-1, EPB41, ZO-2 |
| 3 | Development_Positive regulation of WNT/Beta-catenin signaling in the cytoplasm | 1.421E-06 | IRS-2, IGF-1 receptor, HECTD1, Tcf(Lef), Trabid, USP7,  Tankyrases, Frizzled, ZBED3, Jouberin, BIG2 |
| 4 | Cell adhesion_Endothelial cell contacts by junctional mechanisms | 1.610E-06 | JAM1, MAGI-1(BAIAP1), Plakophilin 4, p120-catenin, Cingulin, ZO-1, ZO-2 |
| 5 | Inhibition of TGF-beta signaling in gastric cancer | 1.610E-06 | p300, SMAD2, MEL1, CBP, Beta-fodrin, ELF3, Sin3A |
| 6 | Cytoskeleton remodeling_Regulation of actin cytoskeleton organization by the kinase effectors of Rho GTPases | 7.266E-06 | BETA-PIX, RhoA-related, RhoB, ERM proteins, PIP5KI,  MyHC, ROCK, PAK, Spectrin |
| 7 | Immune response_IL-7 signaling in T lymphocytes | 2.020E-05 | STAT3, p300, IRS-2, PDK (PDPK1), CBP, Fyn, FOXO3A |
| 8 | Inhibition of Ephrin receptors in colorectal cancer | 5.779E-05 | KLF5, ROCK, Ephrin-A receptors, Ephrin-A receptor 2, TCF7L2 (TCF4), Frizzled |
| 9 | Development_Alpha-1 adrenergic receptors signaling via Cyclic AMP | 6.031E-05 | p300, CBP, PKA-reg (cAMP-dependent), Adenylate cyclase, FOXO3A |
| 10 | Neurophysiological process_Receptor-mediated axon growth repulsion | 8.771E-05 | Plexin A2, ROCK2, ROCK, Fyn, Ephrin-A receptors, Ephrin-A receptor 2, Plexin B1 |
| 11 | G-protein signaling_Regulation of CDC42 activity | 1.016E-04 | p200RhoGAP, Zizimin 1, Fyn, DBS, Frabin, VIL2 (ezrin) |
| 12 | Signal transduction_Activin A signaling regulation | 1.016E-04 | ALK-4, p300, SMAD2, CBP, MECOM, Sin3A |
| 13 | Signal transduction_Non-canonical WNT5A signaling | 1.224E-04 | FZD3, ROCK, G-protein alpha-q, VANGL2, FZD5, FZD9,  NF-AT, TCF7L2 (TCF4), CCDC88C |
| 14 | Development_Early embryonal hypaxial myogenesis | 1.972E-04 | PTCH1, KIF27, STK36, PKA-reg (cAMP-dependent), Adenylate cyclase, Frizzled |
| 15 | Development_Positive regulation of WNT/Beta-catenin signaling in the nucleus | 1.985E-04 | p300, CBP/P300, CBP, Tcf(Lef), CARF, TCF7L2 (TCF4), Frizzled, FOXO3A |
| 16 | ENaC regulation in normal and CF airways | 2.196E-04 | alpha-ENaC, gamma-ENaC, PKA-reg (cAMP-dependent),  Adenylate cyclase, cAMP-GEFI, NEDD4L, G-protein alpha-q/11 |
| 17 | Development_Role of growth factors in the maintenance of embryonic stem cell pluripotency | 2.196E-04 | ALK-4, SMAD2, PDK (PDPK1), IGF-1 receptor, ROCK, FGFR2, c-Fos |
| 18 | Signal transduction_Beta-adrenergic receptors signaling via Cyclic AMP | 2.296E-04 | Adenylate cyclase type VI, PKA-reg (cAMP-dependent),  Adenylate cyclase, NURR1, NUR77, Ca-ATPase2 |
| 19 | Development_Stimulation of differentiation of mouse embryonic fibroblasts into adipocytes by extracellular factors | 2.426E-04 | IRS-2, PDK (PDPK1), IGF-1 receptor, CBP, KLF15,  PKA-reg (cAMP-dependent), Adenylate cyclase, C/EBPdelta |
| 20 | Immune response_IFN-gamma signaling via JAK/STAT and PLC-gamma | 2.474E-04 | STAT3, p300, CBP/P300, Fyn, PKA-reg (cAMP-dependent), TRPC1, C/EBPdelta |
| 21 | Development_Fetal brown fat cell differentiation | 2.778E-04 | IRS-2, PDK (PDPK1), IGF-1 receptor, PPARGC1 (PGC1-alpha),  PKA-reg (cAMP-dependent), Adenylate cyclase, TR-beta |
| 22 | Regulation of Beta-catenin activity in colorectal cancer | 3.113E-04 | eEF2K, p300, KLF5, CBP, G-protein alpha-q,  PKA-reg (cAMP-dependent), Adenylate cyclase |
| 23 | Airway smooth muscle contraction in asthma | 3.113E-04 | MyHC, ROCK, PKA-reg (cAMP-dependent),  Adenylate cyclase, TRPC6, Ca-ATPase2, G-protein alpha-q/11 |
| 24 | GLP-1 in inhibition of beta cell proliferation and function in type 2 diabetes | 4.034E-04 | IRS-2, PDK (PDPK1), IGF-1 receptor, PKA-reg (cAMP-dependent),  HXK4, TCF7L2 (TCF4) |
| 25 | DNA damage_Role of SUMO in p53 regulation | 5.501E-04 | p300, Chk2, RanBP2, CBP |
| 26 | Signal transduction_Adenosine A1 receptor signaling pathway | 5.858E-04 | STAT3, PDK (PDPK1), SFK, PKA-reg (cAMP-dependent),  Adenylate cyclase, CGRP, G-protein alpha-q/11 |
| 27 | Transport_Aldosterone-mediated regulation of ENaC sodium transport | 5.986E-04 | MCR, PDK (PDPK1), alpha-ENaC, gamma-ENaC, NEDD4L |
| 28 | Transcription_Sirtuin6 regulation and functions | 6.460E-04 | SREBP2 (nuclear), SREBP2 precursor, PPARGC1 (PGC1-alpha),  PDK4, SREBP2 (Golgi membrane), HXK4, FOXO3A |
| 29 | Deregulation of canonical WNT signaling in major depressive disorder | 7.002E-04 | FZD3, Tcf(Lef), FZD9, TCF7L2 (TCF4), Frizzled |
| 30 | Muscle contraction_GPCRs in the regulation of smooth muscle tone | 7.062E-04 | MyHC, ROCK, G-protein alpha-q, PKA-reg (cAMP-dependent),  Adenylate cyclase, TRPC6, TRPC1, G-protein alpha-q/11 |
| 31 | Stem cells_Cooperation between Hedgehog, IGF-2 and HGF signaling pathways in medulloblastoma stem cells | 8.141E-04 | PTCH1, PDK (PDPK1), IGF-1 receptor, Tcf(Lef), TCF7L2 (TCF4) |
| 32 | Neuroendocrine transdifferentiation in Prostate Cancer | 8.391E-04 | STAT3, PDK (PDPK1), IGF-1 receptor, Tcf(Lef),  PKA-reg (cAMP-dependent), TCF7L2 (TCF4) |
| 33 | Neurophysiological process_Corticoliberin signaling via CRHR1 | 1.045E-03 | PKA-reg (cAMP-dependent), Adenylate cyclase, NURR1,  NUR77, c-Fos, G-protein alpha-q/11 |
| 34 | PR action in breast cancer: stimulation of metastasis | 1.060E-03 | ROCK2, Tissue factor, IRS-2, ATP1B1 |
| 35 | Signal transduction_PKA signaling | 1.161E-03 | KDELR, PDK (PDPK1), PKA-reg (cAMP-dependent),  Adenylate cyclase, AKAP11, PDE4D |
| 36 | Glucagon-induced glucose upregulation in type 2 diabetes in liver | 1.287E-03 | p300, PPARGC1 (PGC1-alpha), CBP, PKA-reg (cAMP-dependent),  Adenylate cyclase, G-protein alpha-q/11 |
| 37 | Immune response_IL-6 signaling pathway via JAK/STAT | 1.325E-03 | STAT3, p300, CBP, Mcl-1, FOXO3A, c-Fos, C/EBPdelta |
| 38 | Development_Beta adrenergic receptors in brown adipocyte differentiation | 1.412E-03 | PPARGC1 (PGC1-alpha), PKA-reg (cAMP-dependent),  Adenylate cyclase, PRKAR1A, C/EBPdelta |
| 39 | Signal transduction_Calcium-mediated signaling | 1.439E-03 | p300, PPARGC1 (PGC1-alpha), ROCK, MUNC13, NURR1, NUR77, c-Fos |
| 40 | NRF2 regulation of oxidative stress response | 1.571E-03 | MafF, MafK, PDK (PDPK1), NRF2, CBP, Fyn |
| 41 | Development_Negative feedback regulation of WNT/Beta-catenin signaling | 1.602E-03 | LATS2, Tcf(Lef), G-protein alpha-q, TCF7L2 (TCF4), Frizzled |
| 42 | PGE2 pathways in cancer | 1.729E-03 | PDK (PDPK1), Tcf(Lef), PKA-reg (cAMP-dependent),  Adenylate cyclase, NURR1, TCF7L2 (TCF4) |
| 43 | Cytoskeleton remodeling_ACM3 and ACM4 in keratinocyte migration | 1.810E-03 | ROCK2, Adenylate cyclase type VI, G-protein alpha-q,  PKA-reg (cAMP-dependent), G-protein alpha-q/11 |
| 44 | Mitogenic action of ErbB2 in breast cancer | 1.899E-03 | PDK (PDPK1), HUNK, ErbB4, TCF7L2 (TCF4), FOXO3A, c-Fos |
| 45 | CHDI_Correlations from Replication data_Causal network (negative correlations) | 2.284E-03 | CBP, G-protein alpha-q, TCF7L2 (TCF4), Frizzled, Collagen IV |
| 46 | Development_PTHR1 in bone and cartilage development | 2.291E-03 | PTCH1, p300, PKA-reg (cAMP-dependent), Adenylate cyclase,  NURR1, c-Fos, G-protein alpha-q/11 |
| 47 | Signal transduction_Angiotensin II/ AGTR1 signaling via p38, ERK and PI3K | 2.369E-03 | PDK (PDPK1), IGF-1 receptor, KLF5, PPARGC1 (PGC1-alpha),  Fyn, G-protein alpha-q, NUR77, MEKK4(MAP3K4) |
| 48 | Apoptosis and survival_Apoptotic Activin A signaling | 2.523E-03 | ALK-4, SMAD2, PDK (PDPK1), c-Fos |
| 49 | Development_Trophectoderm differentiation | 2.523E-03 | ALK-4, SMAD2, ETS2, FGFR2 |
| 50 | Development_Regulation of lung epithelial progenitor cell differentiation | 2.552E-03 | STAT3, p130, Tcf(Lef), Frizzled, FGFR2 |
