# Supplementary material for: Circadian rhythm-related factors of PER and CRY family genes function as novel therapeutic targets and prognostic biomarkers in lung adenocarcinoma
Source: Aging (Albany NY). 2022 Nov 16;14(22):9056–89. doi: 10.18632/aging.204386 (PMC9740380; doi:10.18632/aging.204386)
Supplement: Supplementary Table 5 [file aging-14-204386-s006.docx]

Supplementary Table 5. Pathway analysis of genes coexpressed with *PER3* from public lung cancer databases using the MetaCore database (with *p*<0.05 set as the cutoff value).

| No. | Map | *p* Value | Network objects from active data |
| --- | --- | --- | --- |
| 1 | Development_Gastrin in cell growth and proliferation | 3.347E-09 | MEKK1(MAP3K1), PDK (PDPK1), PKC-epsilon, PI3K reg class IA (p85),  G-protein alpha-q, EGFR, JNK(MAPK8-10), PI3K reg class IA (p85-alpha), p90Rsk, SOS, IP3 receptor, G-protein alpha-q/11 |
| 2 | NF-AT signaling in cardiac hypertrophy | 6.656E-08 | GAB1, Cardiotrophin-1, p300, CBP, PKC-epsilon, gp130, IL-6 receptor,  Beta-1 adrenergic receptor, PI3K reg class IA, G-protein alpha-q/11, LIF receptor |
| 3 | Immune response_Gastrin in inflammatory response | 1.080E-07 | MEKK1(MAP3K1), PDK (PDPK1), PKC-epsilon, PI3K reg class IA (p85),  G-protein alpha-q, EGFR, JNK(MAPK8-10), SOS, LARG, IP3 receptor, G-protein alpha-q/11 |
| 4 | Immune response_IL-6 signaling pathway via MEK/ERK and PI3K/AKT cascades | 2.645E-07 | GAB1, sIL6-RA, PDK (PDPK1), EGR1, PI3K reg class IA (p85), gp130,  SOS, IL-6 receptor, IP3 receptor, IL6RA, PI3K reg class IA |
| 5 | Androgen receptor activation and downstream signaling in Prostate cancer | 3.402E-07 | GAB1, SPRY2, SPRY1, PDK (PDPK1), TMPRSS2, ER81, gp130, EGFR,  SOS, IL-6 receptor, TLR2, IL6RA, FGFR2 |
| 6 | Development_Gastrin in differentiation of the gastric mucosa | 7.498E-07 | p300, EGR1, CBP, PKC-epsilon, G-protein alpha-q, IP3 receptor, PKC,  G-protein alpha-q/11 |
| 7 | Signal transduction_Angiotensin II/ AGTR1 signaling via p38, ERK and PI3K | 8.215E-07 | PDGF-C, PDK (PDPK1), EGR1, MSK1/2 (RPS6KA5/4), G-protein alpha-q,  EGFR, PI3K reg class IA (p85-alpha), PDGF-D, p90Rsk, SOS, CaMK II delta, ATP7A |
| 8 | Development_Endothelin-1/EDNRA signaling | 8.777E-07 | MEKK1(MAP3K1), FARP2, PKC-epsilon, JNK(MAPK8-10), Adenylate cyclase,  SOS, IP3 receptor, PI3K reg class IA, G-protein alpha-q/11 |
| 9 | Immune response_HMGB1 release from the cell | 9.268E-07 | p300, PDK (PDPK1), CBP, PI3K reg class IA (p85), JNK(MAPK8-10),  TLR2, IP3 receptor, PI3K reg class IA |
| 10 | Proliferative action of Gastrin in gastric cancer | 1.039E-06 | PDK (PDPK1), PI3K reg class IA (p85), G-protein alpha-q,  EGFR, p90Rsk, SOS, IP3 receptor, PKC, G-protein alpha-q/11 |
| 11 | Mitogenic action of ErbB2 in breast cancer | 1.684E-06 | PDK (PDPK1), ER81, SMAD7, PI3K reg class IA (p85),  EGFR, MSK1, ErbB4, SOS, FOXO3A |
| 12 | Neurophysiological process_Thyroliberin signaling | 2.024E-06 | CBP, PKC-epsilon, G-protein alpha-q, Adenylate cyclase,  Tau (MAPT), SOS, MLCK, IP3 receptor, PKC, G-protein alpha-q/11 |
| 13 | Chemotaxis_Lysophosphatidic acid signaling via GPCRs | 2.145E-06 | PDK (PDPK1), EGR1, PKC-epsilon, PI3K reg class IA (p85),  EGFR, JNK(MAPK8-10), MSK1, LARG, MKL2, IP3 receptor, PKC, YAP1 (YAp65), G-protein alpha-q/11 |
| 14 | Apoptosis and survival_NGF/ TrkA PI3K-mediated signaling | 3.329E-06 | GAB1, KIDINS220, PDK (PDPK1), PI3K reg class IA (p85),  MRCKalpha, APC protein, Tau (MAPT), SOS, FOXO3A, p190RhoGAP |
| 15 | Development_Negative regulation of STK3/4 (Hippo) pathway and positive regulation of YAP/TAZ function | 4.055E-06 | PDK (PDPK1), Angiomotin (AMOT), EGFR, JNK(MAPK8-10),  LARG, KIBRA, YAP1 (YAp65), Nephrocystin-4, G-protein alpha-q/11 |
| 16 | IL-6 signaling pathway in lung cancer | 5.436E-06 | GAB1, PDK (PDPK1), gp130, EGFR, IL-6 receptor, IL6RA, PI3K reg class IA |
| 17 | Development_Negative regulation of WNT/Beta-catenin signaling in the cytoplasm | 5.938E-06 | Ankyrin-G, PEG3, Prickle-1, Skp2/TrCP/FBXW, RIPK4,  APC protein, Malin, Laforin, YAP1/TAZ, YAP1 (YAp65) |
| 18 | Development_Thromboxane A2 signaling pathway | 6.668E-06 | MSK1/2 (RPS6KA5/4), PI3K reg class IA (p85),  G-protein alpha-q, EGFR, Adenylate cyclase, IP3 receptor, PKC, PI3K reg class IA |
| 19 | Immune response_Oncostatin M signaling via MAPK | 8.039E-06 | MEKK1(MAP3K1), EGR1, gp130, JNK(MAPK8-10), SOS, LIFR, LIF receptor |
| 20 | Development_TGF-beta receptor signaling | 9.024E-06 | p300, CBP, ER81, SMAD7, Ski, MSK1, SMURF2, SOS |
| 21 | IL-6 signaling in Prostate Cancer | 1.382E-05 | GAB1, PDK (PDPK1), gp130, SOS, IL-6 receptor, IL6RA, PI3K reg class IA |
| 22 | Regulation of Beta-catenin activity in colorectal cancer | 1.588E-05 | eEF2K, p300, CBP, PI3K reg class IA (p85),  G-protein alpha-q, Adenylate cyclase, IP3 receptor, CaSR |
| 23 | Reproduction_Gonadotropin-releasing hormone (GnRH) signaling | 1.597E-05 | MEKK1(MAP3K1), EGR1, PKC-epsilon, JNK(MAPK8-10),  Adenylate cyclase, p90Rsk, SOS, IP3 receptor, G-protein alpha-q/11 |
| 24 | Breast cancer (general schema) | 1.638E-05 | PTCH1, TGF-beta, EGFR, ErbB4, IL-6 receptor, PR (membrane), PR (nuclear) |
| 25 | Development_Neurotrophin family signaling | 1.638E-05 | GAB1, MEKK1(MAP3K1), Sortilin, PI3K reg class IA (p85),  JNK(MAPK8-10), SOS, PI3K reg class IA |
| 26 | Development_VEGF signaling via VEGFR2 - generic cascades | 1.844E-05 | MEKK1(MAP3K1), PDK (PDPK1), p120GAP, MSK1, p90Rsk,  SOS, MLCK, IP3 receptor, PKC, PI3K reg class IA |
| 27 | ESR1 (membrane) 36 kDa isoform signaling in breast cancer | 2.268E-05 | PDK (PDPK1), G-protein alpha-q, EGFR, JNK(MAPK8-10),  SOS, IP3 receptor, PI3K reg class IA |
| 28 | FGF signaling in Prostate Cancer | 2.268E-05 | GAB1, SPRY2, SPRY1, PDK (PDPK1), SOS, FGFR2, PI3K reg class IA |
| 29 | Development_Activation of Erk by ACM1, ACM3 and ACM5 | 2.651E-05 | EGR1, PKC-epsilon, G-protein alpha-q, p120GAP, EGFR, SOS, IP3 receptor |
| 30 | Proliferative action of Gastrin in pancreatic cancer | 2.651E-05 | PDK (PDPK1), PKC-epsilon, PI3K reg class IA (p85),  G-protein alpha-q, SOS, LARG, IP3 receptor |
| 31 | Oxidative stress_ROS-mediated MAPK activation via canonical pathways | 2.667E-05 | GAB1, MEKK1(MAP3K1), PKC-epsilon, EGFR,  JNK(MAPK8-10), SOS, IP3 receptor, CaMK II delta |
| 32 | Signal transduction_Adenosine A1 receptor signaling pathway | 3.403E-05 | PDK (PDPK1), PKC-epsilon, PI3K reg class IA (p85),  EGFR, Adenylate cyclase, IP3 receptor, PKC, G-protein alpha-q/11 |
| 33 | CHDI_DEGs from Replication data_Causal network | 3.727E-05 | MAGI-3, MEKK1(MAP3K1), EGR1, CBP, G-protein alpha-q,  SOS, JNK3(MAPK10), IP3 receptor, PKC |
| 34 | Development_PIP3 signaling in cardiac myocytes | 4.130E-05 | GAB1, PDK (PDPK1), PARD6, p90Rsk, SOS, FOXO3A, PI3K reg class IA |
| 35 | Development_HGF signaling pathway | 4.130E-05 | GAB1, MEKK1(MAP3K1), PDK (PDPK1), EGR1,  JNK(MAPK8-10), SOS, PI3K reg class IA |
| 36 | Development_EGF-induced proliferation of Type C cells in SVZ of adult brain | 4.629E-05 | PTCH1, PDK (PDPK1), APLP2 active fragment, PI3K reg class IA (p85), EGFR, SOS |
| 37 | K-RAS signaling in lung cancer | 4.752E-05 | SPRY2, SPRY1, MEKK1(MAP3K1), PDK (PDPK1),  PI3K reg class IA (p85), EGFR, JNK(MAPK8-10) |
| 38 | Neurogenesis_NGF/ TrkA MAPK-mediated signaling | 5.321E-05 | KIDINS220, EGR1, PDZ-GEF1, PKC-epsilon, MSK1,  RASGRF1, p90Rsk, SOS, SORBS1, IP3 receptor |
| 39 | G-protein signaling_G-Protein alpha-q signaling cascades | 5.526E-05 | PDK (PDPK1), PKC-epsilon, SOS, LARG, IP3 receptor, G-protein alpha-q/11 |
| 40 | Role of neuropeptides in pathogenesis of SCLC | 6.016E-05 | PKC-epsilon, G-protein alpha-q, EGFR, p90Rsk, SOS,  IP3 receptor, PKC, G-protein alpha-q/11 |
| 41 | Development_Growth factors in regulation of oligodendrocyte progenitor cell proliferation | 6.016E-05 | PLP1, PDK (PDPK1), PI3K reg class IA (p85), EGFR,  ErbB4, SOS, PKC, PI3K reg class IA |
| 42 | Pro-inflammatory action of Gastrin in gastric cancer | 6.228E-05 | MEKK1(MAP3K1), PI3K reg class IA (p85), G-protein alpha-q,  EGFR, SOS, LARG, G-protein alpha-q/11 |
| 43 | PI3K signaling in gastric cancer | 6.228E-05 | PDK (PDPK1), PI3K reg class IA (p85), G-protein alpha-q,  EGFR, PI3K reg class IA (p85-alpha), PI3K reg class IA, G-protein alpha-q/11 |
| 44 | Development_Positive regulation of WNT/Beta-catenin signaling in the nucleus | 7.447E-05 | p300, CBP/P300, CBP, CARF, FOXO3A, TLE, ICAT, YAP1 (YAp65) |
| 45 | Development_FGF-family signaling | 8.058E-05 | GAB1, PDK (PDPK1), PKC-epsilon, SOS, IP3 receptor, FGFR2, PI3K reg class IA |
| 46 | G-protein signaling_Proinsulin C-peptide signaling | 8.058E-05 | PDK (PDPK1), PKC-epsilon, PI3K reg class IA (p85),  PI3K reg class IA (p85-alpha), SOS, IP3 receptor, PI3K reg class IA |
| 47 | IGF signaling in HCC | 8.058E-05 | PDK (PDPK1), EGR1, PI3K reg class IA (p85), EGFR, SOS, FOXO3A, PKC |
| 48 | Development_EGFR signaling via PIP3 | 8.292E-05 | GAB1, PDK (PDPK1), EGFR, JNK(MAPK8-10), PI3K reg class IA |
| 49 | Development_Stimulation of differentiation of mouse embryonic fibroblasts into adipocytes by extracellular factors | 9.150E-05 | PDK (PDPK1), CBP, p90RSK2(RPS6KA3), PI3K reg class IA (p85),  Adenylate cyclase, SOS, BMP receptor 2, LIF receptor |
| 50 | Immune response_IL-6 signaling pathway via JAK/STAT | 9.150E-05 | p300, sIL6-RA, MEKK1(MAP3K1), CBP, gp130, IL-6 receptor, FOXO3A, IL6RA |
