# Supplementary material for: Circadian rhythm-related factors of PER and CRY family genes function as novel therapeutic targets and prognostic biomarkers in lung adenocarcinoma
Source: Aging (Albany NY). 2022 Nov 16;14(22):9056–89. doi: 10.18632/aging.204386 (PMC9740380; doi:10.18632/aging.204386)
Supplement: Supplementary Table 6 [file aging-14-204386-s007.docx]

Supplementary Table 6. Pathway analysis of genes coexpressed *CRY1* from public lung cancer databases using the MetaCore database (with *p*<0.05 set as the cutoff value).

| No. | Map | *p* Value | Network objects from active data |
| --- | --- | --- | --- |
| 1 | Development_Positive regulation of WNT/Beta-catenin signaling in the cytoplasm | 8.294E-08 | TGT, GSK3 alpha/beta, PP2C alpha, ITGB1, Trabid, PP1-cat,  JNK(MAPK8-10), SMAD4, Frizzled, AKT(PKB), BIG2 |
| 2 | Signal transduction_Non-canonical WNT5A signaling | 1.660E-06 | MLCP (cat), FZD6, MLCP (reg), ATF-2, ROCK, RYK,  JNK(MAPK8-10), TAK1(MAP3K7), Calcineurin A (catalytic), PLC-beta |
| 3 | Oxidative stress_ROS-induced cellular signaling | 3.050E-06 | AMPK alpha subunit, SENP1, IKK-alpha, JNK1(MAPK8),  ADAM17, JNK(MAPK8-10), Sirtuin1, GSK3 beta, IRP2, SAE2, AKT(PKB) |
| 4 | Cell cycle_Influence of Ras and Rho proteins on G1/S Transition | 3.661E-06 | ROCK2, MLCP (cat), MLCP (reg), ATF-2, IKK-alpha,  JNK1(MAPK8), GSK3 beta, AKT(PKB) |
| 5 | Signal transduction_Calcium-mediated signaling | 4.512E-06 | MLCP (cat), AMPK alpha subunit, MLCP (reg), ATF-2,  PPARGC1 (PGC1-alpha), ROCK, JNK(MAPK8-10), Calcineurin A (catalytic), AKT(PKB) |
| 6 | Immune response_IL-11 signaling pathway via MEK/ERK and PI3K/AKT cascades | 2.180E-05 | YES, ADAM10, SFK, IKK-alpha, SHP-2, GSK3 beta, ATF-1, AKT(PKB) |
| 7 | IgE- and MGF-induced Lyn-mediated production of cytokines and arachidonic acid metabolites in lung mast cells in asthma | 2.512E-05 | MGF, c-Kit, IKK-alpha, JNK1(MAPK8), GSK3 beta,  Calcineurin A (catalytic), AKT(PKB) |
| 8 | Development_The role of GDNF ligand family/ RET receptor in cell survival, growth and proliferation | 3.396E-05 | GDNF, ITGB1, IKK-alpha, ROCK, SHP-2, JNK1(MAPK8),  ATF-1, CREM (activators), AKT(PKB) |
| 9 | Aberrant B-Raf signaling in melanoma progression | 4.736E-05 | AMPK alpha subunit, Rictor, ITGB1, ROCK,  JNK(MAPK8-10), AKT3, AKT(PKB) |
| 10 | Noise-induced hair cell death and spiral ganglion neuron degeneration | 4.769E-05 | ROCK2, AMPK alpha subunit, GDNF, ERM proteins,  Apaf-1, ROCK, JNK(MAPK8-10), AMPK beta subunit, Calcineurin A (catalytic) |
| 11 | Signal transduction_BMP signaling via BMPR1A and BMPR1B receptors | 5.603E-05 | SMAD9 (SMAD8), ID2, ATF-2, BMPR1A,  TAK1(MAP3K7), SMAD4 |
| 12 | Dual role of BMP signaling in gastric cancer | 6.460E-05 | SMAD9 (SMAD8), IKK-alpha, BMPR1A, SMAD4, AKT(PKB) |
| 13 | Signal transduction_Angiotensin II/ AGTR1 signaling via RhoA and JNK | 6.640E-05 | ROCK2, MLCP (cat), MLCP (reg), ATF-2,  ROCK, ECT2, JNK(MAPK8-10), PLC-beta1 |
| 14 | TGF-beta signaling via kinase cascades in breast cancer | 6.710E-05 | ATF-2, ITGB1, IKK-alpha, ADAM17, JNK(MAPK8-10),  TAK1(MAP3K7), AKT(PKB) |
| 15 | Development_Negative regulation of WNT/Beta-catenin signaling in the cytoplasm | 9.503E-05 | GSK3 alpha/beta, CXXC4, DP1, Skp2/TrCP/FBXW,  JNK1(MAPK8), PP1-cat, LATS1, Frizzled |
| 16 | Immune response_Mast cell proliferation, differentiation and survival | 1.613E-04 | MGF, c-Kit, SHP-2, JNK(MAPK8-10), AKT(PKB) |
| 17 | Immune response_BAFF-induced non-canonical NF-kB signaling | 1.613E-04 | IKK-alpha, ZFP91, Skp2/TrCP/FBXW, E2N(UBC13), NEDD8 |
| 18 | Fibroblast differentiation to myofibroblasts in asthmatic airways | 1.896E-04 | ITGB1, JNK(MAPK8-10), TAK1(MAP3K7), SMAD4, AKT(PKB) |
| 19 | Neurophysiological process_HTR2A signaling in the nervous system | 2.074E-04 | SFK, MUPP1, Calcineurin A (catalytic), PLC-beta1, AKT(PKB), PLC-beta |
| 20 | Inhibition of LKB1 / AMPK signaling in breast cancer | 2.215E-04 | PPARGC1 (PGC1-alpha), TFAM, GSK3 beta,  AMPK alpha 1 subunit, AKT(PKB) |
| 21 | Mechanisms of drug resistance in SCLC | 2.238E-04 | MGF, ITGB1, c-Kit, Apaf-1, IKK-alpha, GSK3 beta, AKT(PKB) |
| 22 | Development_Role of PKR1 and ILK in cardiac progenitor cells | 2.573E-04 | ITGB1, c-Kit, GSK3 beta, AKT(PKB), PLC-beta |
| 23 | Development_BMP signaling in cardiac myogenesis | 2.974E-04 | ID2, ATF-2, BMPR1A, TAK1(MAP3K7), SMAD4 |
| 24 | Signal transduction_HTR2A signaling outside the nervous system | 3.165E-04 | MLCP (cat), MLCP (reg), PFKM, ROCK, ADAM17, AKT(PKB), PLC-beta |
| 25 | Extracellular matrix-regulated proliferation of airway smooth muscle cells in asthma | 3.420E-04 | ATF-2, JNK1(MAPK8), JNK(MAPK8-10), TAK1(MAP3K7), SMAD4 |
| 26 | Tau pathology in Alzheimer disease | 3.938E-04 | eIF2AK3, GSK3 alpha/beta, JNK(MAPK8-10),  GSK3 beta, Calcineurin A (catalytic), PP2C |
| 27 | Development_WNT/Beta-catenin signaling in the cytoplasm | 3.938E-04 | GSK3 alpha/beta, GSK3 beta, Frizzled, IPMK, PLC-beta1, AKT(PKB) |
| 28 | Apoptosis and survival_NGF/ TrkA PI3K-mediated signaling | 4.046E-04 | MLCP (cat), MLCP (reg), ROCK, GSK3 beta, PARD3,  Calcineurin A (catalytic), AKT(PKB) |
| 29 | Role of activation of WNT signaling in the progression of lung cancer | 4.046E-04 | FZD6, JNK1(MAPK8), Sirtuin1, GSK3 beta, BMI-1, Frizzled, SUZ12 |
| 30 | Signal transduction_BMP signaling via ALK-1 and ALK-2 receptors | 4.431E-04 | SMAD9 (SMAD8), ID2, BMPR1A, SMAD4 |
| 31 | Down-regulation of mast cell functions through ITIM-containing inhibitory receptors in asthma | 4.463E-04 | CD200 (OX-2), MGF, c-Kit, OX-2 receptor 1, SHP-2 |
| 32 | Development_MAG, Reticulon 4 and OMgp in inhibition of neurite outgrowth | 4.734E-04 | MLCP (cat), MLCP (reg), ROCK, SHP-2, ADAM17,  GSK3 beta, AKT(PKB) |
| 33 | Signal transduction_Angiotensin II signaling via Beta-arrestin | 4.788E-04 | MLCP (cat), MLCP (reg), ROCK, DGK, GSK3 beta, AKT(PKB) |
| 34 | Development_Noncanonical WNT signaling in cardiac myogenesis | 5.066E-04 | ROCK2, ATF-2, JNK(MAPK8-10), SMAD4, PLC-beta |
| 35 | Inhibition of GSK3 beta by lithium in major depressive disorder | 5.342E-04 | PP1-cat, GSK3 beta, SMAD4, AKT(PKB) |
| 36 | CHDI_DEGs from Replication data_Causal network | 5.513E-04 | IKK-alpha, PP1-cat, GSK3 beta, Calcineurin A (catalytic),  Frizzled, PLC-beta1, AKT(PKB) |
| 37 | Apoptosis and survival_Regulation of apoptosis by mitochondrial proteins | 5.606E-04 | DNM1L (DRP1), ATF-2, Apaf-1, JNK(MAPK8-10), OPA1,  Calcineurin A (catalytic), PP2C, Mitofusin 1 |
| 38 | Development_BMP7 in brown adipocyte differentiation | 5.729E-04 | ATF-2, PPARGC1 (PGC1-alpha), TFAM, BMPR1A, TAK1(MAP3K7) |
| 39 | Oxidative stress_ROS-mediated MAPK activation via canonical pathways | 6.322E-04 | SFK, SHP-2, JNK1(MAPK8), ADAM17, JNK(MAPK8-10), MAP3K2 (MEKK2) |
| 40 | Stem cells_NOTCH1-induced self-renewal of glioblastoma stem cells | 6.455E-04 | SMAD9 (SMAD8), DLL1, BMPR1A, ADAM17, SMAD4 |
| 41 | Development_WNT/Beta-catenin and NOTCH in induction of osteogenesis | 6.455E-04 | DLL1, ADAM17, GSK3 beta, SMAD4, Frizzled |
| 42 | Stem cells_Response to hypoxia in glioblastoma stem cells | 6.455E-04 | SMAD9 (SMAD8), DLL1, BMPR1A, ADAM17, SMAD4 |
| 43 | Development_Astrocyte differentiation from adult stem cells | 6.455E-04 | SMAD9 (SMAD8), ID2, BMPR1A, SHP-2, SMAD4 |
| 44 | Signal transduction_MIF signaling pathway | 6.911E-04 | SFK, JNK(MAPK8-10), GSK3 beta, ACKR3, AKT(PKB), PLC-beta |
| 45 | Immune response_B cell antigen receptor (BCR) pathway | 7.173E-04 | GSK3 alpha/beta, ATF-2, IKK-alpha, GSK3 beta,  TAK1(MAP3K7), Calcineurin A (catalytic), PIP5KIII, AKT(PKB) |
| 46 | Development_Negative regulation of STK3/4 (Hippo) pathway and positive regulation of YAP/TAZ function | 7.540E-04 | MLCP (cat), MLCP (reg), Mol1b, LATS1, JNK(MAPK8-10), PARD3 |
| 47 | Role of TNF-alpha in type 2 diabetes in skeletal muscle cells | 8.110E-04 | PP2C alpha, AMPK alpha subunit, PPARGC1 (PGC1-alpha),  JNK1(MAPK8), ADAM17 |
| 48 | Development_Growth hormone signaling via PI3K/AKT and MAPK cascades | 8.110E-04 | ATF-2, SHP-2, ADAM17, GSK3 beta, AKT(PKB) |
| 49 | Neuroprotective action of lithium | 8.213E-04 | ATF-2, JNK1(MAPK8), PP1-cat, GSK3 beta, Calcineurin A (catalytic), Frizzled |
| 50 | Regulation of metabolism_Role of Adiponectin in regulation of metabolism | 9.047E-04 | AMPK alpha subunit, PPARGC1 (PGC1-alpha), TFAM, Sirtuin1, ACADM |
