# Supplementary material for: Circadian rhythm-related factors of PER and CRY family genes function as novel therapeutic targets and prognostic biomarkers in lung adenocarcinoma
Source: Aging (Albany NY). 2022 Nov 16;14(22):9056–89. doi: 10.18632/aging.204386 (PMC9740380; doi:10.18632/aging.204386)
Supplement: Supplementary Table 7 [file aging-14-204386-s008.docx]

Supplementary Table 7. Pathway analysis of genes coexpressed with *CRY2* from public lung cancer databases using the MetaCore database (with *p*<0.05 set as the cutoff value).

| No. | Map | *p* Value | Network objects from active data |
| --- | --- | --- | --- |
| 1 | Cytoskeleton remodeling_Regulation of actin cytoskeleton nucleation and polymerization by Rho GTPases | 2.637E-08 | RhoA, RhoA-related, RhoD, DRF, RhoF (Rif), Rac1-related,  Cdc42 subfamily, RhoC, BAIAP2, RhoB, TC10, PIP5KI, FNBP1, Gelsolin |
| 2 | Development_Regulation of lung epithelial progenitor cell differentiation | 4.253E-07 | SMAD9 (SMAD8), p130, Beta-catenin, SP-C, SP-A, SHH,  FGFR2, GATA-6, WNT, SP-B, FOXP1, Napsin A |
| 3 | Cytoskeleton remodeling_Regulation of actin cytoskeleton organization by the kinase effectors of Rho GTPases | 6.410E-07 | RhoA, RhoA-related, WRCH-1, Alpha-actinin, Rac1-related,  Alpha adducin, Cdc42 subfamily, RhoJ, RhoC, RhoB, ERM proteins, TC10, PIP5KI, MyHC |
| 4 | Development_Oligodendrocyte differentiation from adult stem cells | 8.214E-07 | PLP1, MCT8, TR-alpha, SMAD9 (SMAD8), FGFR3, EDNRB,  SHH, SMAD6, PTCH1, CNTN6, SMAD7, p90Rsk, TR-beta |
| 5 | NF-AT signaling in cardiac hypertrophy | 2.792E-06 | AGTR1, MEF2A, gp130, HDAC5, Beta-1 adrenergic receptor,  PI3K reg class IA, G-protein alpha-q/11, GAB1, Cardiotrophin-1, PKC-epsilon, Troponin I, cardiac, IL-6 receptor, CAMTA2, LIF receptor |
| 6 | Breast cancer (general schema) | 3.357E-06 | PTHR1, Androgen receptor, ErbB4, SHH, Ihh, PTCH1,  TGF-beta receptor type II, IL-6 receptor, PR (membrane), GHR, PR (nuclear) |
| 7 | Development_Role of G-CSF in hematopoietic stem cell mobilization | 4.553E-05 | C5aR, Leukocyte elastase, S1P1 receptor, Cathepsin G,  C5a, Cathepsin K, Carboxypeptidase M |
| 8 | ENaC regulation in normal and CF airways | 4.702E-05 | Dopamine D1A receptor, gamma-ENaC, PKA-reg (cAMP-dependent),  WWP2, EDNRB, Beta-2 adrenergic receptor, cAMP-GEFI,  PI3K reg class IA, G-protein alpha-q/11, Adenylate cyclase, NEDD4L |
| 9 | Muscle contraction_GPCRs in the regulation of smooth muscle tone | 5.296E-05 | AGTR1, RhoA, G-protein alpha-q, PKA-reg (cAMP-dependent),  Beta-2 adrenergic receptor, TRPC6, Adenosine A2b receptor, TRPC1, G-protein alpha-q/11, MELC, MyHC, Adenylate cyclase, Alpha-1A adrenergic receptor, IP3 receptor |
| 10 | Signal transduction_Beta-adrenergic receptors signaling via Cyclic AMP | 7.741E-05 | AHNAK, KCNQ1, PKA-reg (cAMP-dependent), Phospholemman,  Beta-2 adrenergic receptor, Beta-1 adrenergic receptor,  Troponin I, cardiac, Ryanodine receptor 2, Adenylate cyclase |
| 11 | Regulation of Beta-catenin activity in colorectal cancer | 8.020E-05 | eEF2K, Beta-catenin, PI3K reg class IA (p85),  G-protein alpha-q, PKA-reg (cAMP-dependent), CaSR, PKC-delta,  PDCD4, Axin, Adenylate cyclase, IP3 receptor |
| 12 | Induction of mucin secretion in airway goblet cells by purinergic receptors | 9.499E-05 | PP2A regulatory, KCNQ1, MUNC13-2, G-protein alpha-q/11,  HSP70, MUNC18, PKC-delta, PKC-epsilon, MUNC13, MYO5C, IP3 receptor |
| 13 | Neurophysiological process_ACM regulation of nerve impulse | 1.002E-04 | RhoA, G-protein alpha-q, PKA-reg (cAMP-dependent),  CACNA1H, TRPC6, PKC, G-protein alpha-q/11, ACM1, CACNA1G, IP3 receptor |
| 14 | Development_Endothelial differentiation during embryonic development | 1.712E-04 | Angiopoietin 1, Beta-catenin, COUP-TFII, FGFR3,  TIE2, SHH, Ihh, PKC, PI3K reg class IA, WNT |
| 15 | G-protein signaling_Rap2B regulation pathway | 1.871E-04 | MR-GEF, Beta-1 adrenergic receptor, PDZ-GEF1, PLC-epsilon |
| 16 | Immune response_IL-11 signaling via JAK/STAT | 2.038E-04 | IL11RA, Leukocyte elastase, gp130, IL-11 receptor,  A2M, sIL11-RA, SP-B, CYP19 |
| 17 | Development_Gastrin in cell growth and proliferation | 2.088E-04 | Beta-catenin, PI3K reg class IA (p85), G-protein alpha-q,  JNK(MAPK8-10), PI3K reg class IA (p85-alpha),  G-protein alpha-q/11, PDK (PDPK1), PKC-delta, PKC-epsilon, p90Rsk, IP3 receptor |
| 18 | Regulation of lipid metabolism_Regulation of fatty acid synthase activity in hepatocytes | 2.281E-04 | RXRA, SREBP1 precursor, SREBP1 (nuclear), SREBP1 (Golgi membrane),  S1P, TR-beta |
| 19 | Immune response_Antigen presentation by MHC class II | 2.345E-04 | MHC class II alpha chain, RhoA, Cathepsin F, MHC class II, PIP5K1A,  JNK(MAPK8-10), FCGRT, ARHGEF2, PKC,  RILP (Rab interacting lysosomal protein), MHC class II beta chain, ARL14, PDK (PDPK1), PKC-delta, RhoB, CLEC10A |
| 20 | Cell adhesion_Histamine H1 receptor signaling in the interruption of cell barrier integrity | 3.083E-04 | RhoA, Occludin, Beta-catenin, Alpha-actinin,  G-protein alpha-q/11, MELC, PKC-delta, Alpha-catenin, IP3 receptor |
| 21 | Development_Beta-adrenergic receptor-induced regulation of ERK | 4.339E-04 | PKA-reg (cAMP-dependent), Beta-2 adrenergic receptor,  cAMP-GEFI, Beta-1 adrenergic receptor, PDK (PDPK1),  PDZ-GEF1, PLC-epsilon, Adenylate cyclase, IP3 receptor |
| 22 | Regulation of angiogenesis in prostate cancer | 4.339E-04 | AGTR1, Angiopoietin 1, Androgen receptor, PI3K reg class IA (p85),  TIE2, VEGF-D, Collagen IV, PDK (PDPK1), TGF-beta receptor type II |
| 23 | Development_PTHR1 in bone and cartilage development | 4.344E-04 | PTHR1, RhoA, Beta-catenin, MEF2A, PP2A regulatory,  PKA-reg (cAMP-dependent), Ihh, PKC, G-protein alpha-q/11,  PTCH1, Adenylate cyclase, MKP-1 |
| 24 | Ca(2+)-dependent NF-AT signaling in cardiac hypertrophy | 4.424E-04 | AGTR1, MEF2A, G-protein alpha-q, PKA-reg (cAMP-dependent),  MEF2, TRPC6, Beta-1 adrenergic receptor, G-protein alpha-q/11,  Alpha-1A adrenergic receptor, IP3 receptor |
| 25 | Development_Noncanonical WNT signaling in cardiac myogenesis | 4.605E-04 | RhoA, G-protein alpha-q, NF-AT4(NFATC3), JNK(MAPK8-10),  PKC, PKC-delta, Troponin I, cardiac, WNT11 |
| 26 | Signal transduction_mTORC2 downstream signaling | 4.808E-04 | PKC-zeta, RhoA, Beta-catenin, PKA-reg (cAMP-dependent),  OSR1, SREBP1 (nuclear), PKC, PKC-delta, Adenylate cyclase type IX,  FOXO3A, NEDD4L |
| 27 | K-RAS signaling in lung cancer | 5.109E-04 | Beta-catenin, PI3K reg class IA (p85), JNK(MAPK8-10),  Amphiregulin, BTG2, SPRY2, PDK (PDPK1), RhoB, PDCD4 |
| 28 | CHDI_Correlations from Replication data_Causal network (negative correlations) | 6.635E-04 | RhoA, Beta-catenin, G-protein alpha-q, PKC, Collagen IV,  WNT, Axin, LPP3 |
| 29 | IL-6 signaling in Prostate Cancer | 6.635E-04 | Androgen receptor, gp130, BMX, PI3K reg class IA, GAB1,  PDK (PDPK1), IL-6 receptor, IL6RA |
| 30 | Development_Thromboxane A2 signaling pathway | 6.986E-04 | RhoA, Beta-catenin, PI3K reg class IA (p85), G-protein alpha-q,  PKA-reg (cAMP-dependent), PKC, PI3K reg class IA,  Adenylate cyclase, IP3 receptor |
| 31 | Development_Positive regulation of STK3/4 (Hippo) pathway and negative regulation of YAP/TAZ function | 7.019E-04 | RhoA, Dopamine D1A receptor, Beta-catenin,  PKA-reg (cAMP-dependent), Beta-2 adrenergic receptor,  KIBRA, Axin, Adenylate cyclase, Alpha-catenin, Alpha-1 catenin, LIF receptor |
| 32 | Signal transduction_Calcium-mediated signaling | 7.921E-04 | CABIN1, RhoA, PPARGC1 (PGC1-alpha), JNK(MAPK8-10),  Myocardin, MEF2, PKC, HDAC5, MYH11, MUNC13, IP3 receptor |
| 33 | Signal transduction_PKA signaling | 8.116E-04 | Androgen receptor, PP2A regulatory, LBC,  PKA-reg (cAMP-dependent), PHK beta, p90RSK1,  PDK (PDPK1), Troponin I, cardiac, Adenylate cyclase |
| 34 | Putative pathways for stimulation of fat cell differentiation by Bisphenol A | 8.180E-04 | Androgen receptor, SREBP1 precursor, Beta-catenin,  PPARGC1 (PGC1-alpha), PI3K reg class IA, PDK (PDPK1), LPL |
| 35 | Development_Negative regulation of STK3/4 (Hippo) pathway and positive regulation of YAP/TAZ function | 8.813E-04 | AGTR1, RhoA, JNK(MAPK8-10), ARHGEF2, KIBRA,  Nephrocystin-4, G-protein alpha-q/11, PDK (PDPK1), LIMD1, ASPP1 |
| 36 | Reproduction_Gonadotropin-releasing hormone (GnRH) signaling | 8.917E-04 | PKA-reg (cAMP-dependent), JNK(MAPK8-10), PER1,  HDAC5, G-protein alpha-q/11, PKC-delta, PKC-epsilon,  Adenylate cyclase, p90Rsk, IP3 receptor, MKP-1 |
| 37 | CHDI_Correlations from Discovery data_Causal network | 8.917E-04 | Dopamine D1A receptor, Beta-catenin, PI3K reg class IA (p85),  G-protein alpha-q, MEF2, HDAC5, WNT, Axin, p90Rsk, IP3 receptor, TSC-22 |
| 38 | IGF-1 receptor/EGFR cooperation in lung cancer | 9.149E-04 | PKC-zeta, Amphiregulin, PI3K reg class IA, PDK (PDPK1), PKC-delta, p90Rsk |
| 39 | Cell adhesion_Endothelial cell contacts by non-junctional mechanisms | 9.149E-04 | MAGI-1(BAIAP1), Beta-catenin, Alpha-actinin, Collagen IV, ESAM, Alpha-catenin |
| 40 | Signal transduction_Additional pathways of NF-kB activation (in the cytoplasm) | 9.391E-04 | PKC-zeta, PKA-reg (cAMP-dependent), p90RSK1,  PI3K reg class IA, PDK (PDPK1), MAP3K3, PKC-delta,  PKC-epsilon, Adenylate cyclase |
| 41 | Immune response_IL-6 signaling pathway via MEK/ERK and PI3K/AKT cascades | 1.001E-03 | PI3K reg class IA (p85), gp130, p90RSK1,  PI3K reg class IA, GAB1, sIL6-RA, PDK (PDPK1),  PKC-delta, IL-6 receptor, IP3 receptor, IL6RA |
| 42 | Proinflammatory cytokine production by Th17 cells in asthma | 1.082E-03 | C5aR, C5, MHC class II, JNK(MAPK8-10), ROR-alpha,  CRTH2, ROR-gamma, C5a, IL-6 receptor |
| 43 | Translation_Translation regulation by Alpha-1 adrenergic receptors | 1.082E-03 | eEF2K, RhoA, G-protein alpha-q, PI3K reg class IA (p85-alpha),  PKC-delta, Alpha-1D adrenergic receptor, PKC-epsilon,  Alpha-1A adrenergic receptor, IP3 receptor |
| 44 | NF-kB-, AP-1- and MAPKs-mediated proinflammatory cytokine production by eosinophils in asthma | 1.097E-03 | Leukocyte elastase, Cathepsin G, JNK(MAPK8-10), STAT6,  Tryptase, TSLP, C5a, IL-33 |
| 45 | Transport_cAMP/ Ca(2+)-dependent Insulin secretion | 1.284E-03 | PKA-reg (cAMP-dependent), cAMP-GEFI, PKC-epsilon,  PLC-epsilon, Ryanodine receptor 2, VAMP2, IP3 receptor, CACNA1D |
| 46 | Development_WNT/Beta-catenin signaling in the cytoplasm | 1.422E-03 | Axin2, RhoA, Beta-catenin, G-protein alpha-q, IP3KB, FRAT1, WNT, MACF1, Axin |
| 47 | Development_Fetal brown fat cell differentiation | 1.422E-03 | PKC-zeta, SREBP1 precursor, PPARGC1 (PGC1-alpha),  PI3K reg class IA (p85), PKA-reg (cAMP-dependent),  SREBP1 (nuclear), PDK (PDPK1), Adenylate cyclase, TR-beta |
| 48 | Production and activation of TGF-beta in airway smooth muscle cells | 1.436E-03 | AGTR1, RhoA, Leukocyte elastase, TGF-beta receptor type III (betaglycan),  Beta-tryptase 2, Tryptase, TGF-beta receptor type II |
| 49 | Cell adhesion_Endothelial cell contacts by junctional mechanisms | 1.438E-03 | MAGI-1(BAIAP1), Occludin, Beta-catenin, JAM2, Alpha-actinin, Alpha-catenin |
| 50 | Cell adhesion_Classical cadherin-mediated cell adhesion | 1.438E-03 | MAGI-1(BAIAP1), Beta-catenin, Alpha-actinin, BAIAP2, PDZ-GEF1, Alpha-catenin |
